# Bi‐Shell Valve for Fast Actuation of Soft Pneumatic Actuators via Shell Snapping Interaction

**DOI:** 10.1002/advs.202100445

**Published:** 2021-06-01

**Authors:** Chuan Qiao, Lu Liu, Damiano Pasini

**Affiliations:** ^1^ Department of Mechanical Engineering McGill University Montreal Quebec H3A 0C3 Canada

**Keywords:** actuation methods, elastic instabilities, fast actuation, snap‐through buckling, soft pneumatic actuators, soft robots, soft valves

## Abstract

Rapid motion in soft pneumatic robots is typically achieved through actuators that either use a fast volume input generated from pressure control, employ an integrated power source, such as chemical explosions, or are designed to embed elastic instabilities in the body of the robot. This paper presents a bi‐shell valve that can fast actuate soft actuators neither relying on the fast volume input provided by pressure control strategies nor requiring modifications to the architecture of the actuator. The bi‐shell valve consists of a spherical cap and an imperfect shell with a geometrically tuned defect that enables shell snapping interaction to convert a slowly dispensed volume input into a fast volume output. This function is beyond those of current valves capable to perform fluidic flow regulation. Validated through experiments, the analysis unveils that the spherical cap sets the threshold of the snapping pressure along with the upper bounds of volume and energy output, while the imperfect shell interacts with the cap to store and deliver the desired output for rapid actuation. Geometry variations of the bi‐shell valve are provided to show that the concept is versatile. A final demonstration shows that the soft valve can quickly actuate a striker.

## Graphical abstract

A bi‐shell valve leveraging shell snap‐through buckling and interaction is proposed for the rapid actuation of pneumatic soft actuators. A demonstrative experiment shows that the actuation time can be reduced from 13 s (without the valve) to 0.16 s (with the valve). The concept here introduced offers a new function that is unattainable by existing soft valves.

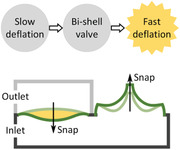

## 1 Introduction

Pneumatic soft robotics is a rapidly evolving field of research that promises to expand the scope of current robotic applications. Distinct from classical rigid body robots, their pneumatic soft counterparts are typically fabricated from soft materials, e.g., elastomers,^[^ ^1^, ^2^ ^]^ that can undergo large deformations to accomplish complex tasks. A diverse range of input sources are typically used to drive their motion; some resort to internal pressurized air,^[^ ^3^, ^4^, ^5^ ^]^ and others to external propellers, such as roller modules^[^ ^6^ ^]^ or moveable bodies.^[^ ^7^ ^]^ Functions that have been realized span a broad spectrum of motion, from locomotion, including galloping,^[^ ^8^ ^]^ swimming,^[^ ^9^ ^]^ crawling,^[^ ^10^ ^]^ and climbing,^[^ ^11^, ^12^, ^13^ ^]^ to manipulation, such as gripping,^[^ ^14^, ^15^ ^]^ stirring,^[^ ^16^ ^]^ and swallowing.^[^ ^7^ ^]^ An advantage offered by soft robots is that their elastic modulus is similar to that of soft biological tissues, imparting in them the ability to easily adapt to the local profile of adjacent objects, and making them suitable for applications that involve delicate interactions with humans,^[^ ^17^, ^18^ ^]^ the handling of fragile objects^[^ ^14^ ^]^ and the adaptation to unknown environments.^[^ ^5^ ^]^ Pneumatic soft robots do not always require a complex control system of actuators, sensors and control algorithms, as rigid body robots typically do; rather their working principle is to achieve control mainly in a passive way, through either the tuning of the robot body compliance ^[^ ^1^, ^19^, ^20^ ^]^ or the integration of a complex internal fluidic circuit.^[^ ^21^, ^22^, ^23^, ^24^ ^]^ Others of their advantages include low mass and cost, high cycle life, availability at small length scales, and damage resistance to impact.^[^ ^2^, ^25^, ^26^, ^27^ ^]^

Key to actuating fluidic soft robots is to have an effective strategy that can power motion and enable complex movements. The most widely used method is pressure control, applied successfully to trigger motion of soft robots of various size.^[^ ^4^, ^26^, ^28^ ^]^ With this strategy, actuation can be rapid and inflation can occur at high speed through the delivery of high pressure^[^ ^4^, ^15^ ^]^ low viscosity air, and/or by further optimizing the actuator design with the outcome of reducing the amount of air required for actuation.^[^ ^26^ ^]^ While effective, pressure‐controlled actuation has a twofold drawback. First, for pressure generation and control, it requires a bulky pneumatic system that typically includes a pressure supply (e.g., air pumps or compressed gas tanks) and a set of hard valves (e.g., pressure regulators and solenoid valves). Second, fine control over the change in volume is difficult to achieve. For a soft robot, this might pose a problem. If motion is driven by the emergence of unstable events, the soft robot might lose its capacity to function properly. For example, a spherical cap embedded into a soft robot that is driven by external pressure will snap from the initial to its fully everted configuration, leaving no chance to access any intermediate states of deformation;^[^ ^29^ ^]^ if motion requires operation at these states, the soft robot will inevitably fail to do so.

An alternative to pressure‐controlled actuation is volume control. This strategy allows for direct adjustment of the volume change. For example, a syringe pump can be used to dispense a precise volume of a fluid into the soft robot, and its pressure‐volume response can be registered.^[^ ^29^, ^30^, ^31^, ^32^, ^33^ ^]^ Besides this advantage, controlling the output volume avoids any jumps in displacement that a pressure control strategy would impose upon snap‐through buckling. Volume control strategies used so far, however, have a common drawback; they are unable to drive fast actuation, a limitation ascribed to the limited flow rate that a syringe pump can typically deliver. Only a handful of attempts have been successful to circumvent this issue, and all of them have been devoted to the design of actuators. Their strategy has been to integrate snap‐through instability of spherical caps^[^ ^31^ ^]^ or balloons^[^ ^32^ ^]^ into the architecture of the actuator.

Besides pressure‐controlled and volume‐controlled actuation, other actuation methods used in the literature resort to external manipulation,^[^ ^6^, ^7^ ^]^ dielectric elastomers,^[^ ^34^, ^35^, ^36^, ^37^ ^]^ evaporation of low‐boiling point fluids,^[^ ^38^ ^]^ chemical decomposition,^[^ ^23^ ^]^ and explosive chemical reaction.^[^ ^39^, ^40^, ^41^, ^42^ ^]^ Their application to soft robots has so far shown to be limited due to the additional components they require, including external mechanisms for manipulation, electrical circuits for dielectric elastomers, heaters for low‐boiling point fluids, and integrated systems for chemical decomposition and combustion.

Pneumatic soft robots typically resort to elements other than actuators to operate. One of them is valves. Their function so far has been other than that of actuators. Current valves can control the fluid flow spreading throughout the body of a soft robot. Some concepts comprise rigid elements that can provide a simple and unambiguous control of a fluid flow. Others achieve this function by engaging elastic instabilities, e.g., wrinkling, snapping, and creasing, in their constituents. Sources used to initiate elastic instabilities include air pressure supply, external force, and viscous flux through unstable‐arch channels. In all cases, the fluid‐control function these valves perform is binary, switching between two distinct states. For example, a spherical cap embedded in the valve has been shown effective to snap from one position to either block or unlock the flow in its internal tubes.^[^ ^29^ ^]^ Another valve architecture exploits the snapping of an elastic arch embedded in a fluid flow to act as a passive microfluidic fuse that regulates the flow present in its rigid‐walled channels.^[^ ^43^ ^]^ Manifold applications exist for soft valves ranging from soft ring oscillators^[^ ^44^ ^]^ to pneumatic logic gates^[^ ^22^ ^]^ and soft kink valves.^[^ ^45^ ^]^ All the existing implementations can only either fully (on‐off control) or partially (binary flux control) block a fluid flow for continuous operation, but cannot provide an impulse for fast actuation under volume control.

In this work, we introduce a bi‐shell valve that can provide volume‐controlled rapid actuation to soft actuators. The valve does not resort to the fast volume input typically generated through pressure control^[^ ^4^, ^15^ ^]^ strategies, nor to any modifications to the body of the actuator that require chemical explosion^[^ ^39^, ^40^, ^41^, ^42^ ^]^ or elastic instability.^[^ ^31^, ^32^, ^36^, ^37^ ^]^ The valve engages snapping and shell interaction to generate a fast volume output upon a slow volume input. Our bi‐shell valve can thus perform a function that is unattainable by existing soft valves. It has the following merits for soft robotics: i) Ready for use with volume control: Common soft actuators can directly use this valve to achieve rapid motion under volume control, without any additional modifications to the body of the actuator. ii) Output performance attuning: The amount of fast volume output can be set in a fully passive way by simply programming the geometry of the constituent shells and their defects to maximize the valve performance and satisfy the functional requirements of a given soft robot. iii) Retainment of pre‐snapping geometry: The volume output of the valve is negligible before snapping, thus enabling the soft actuator connected to our valve to preserve its initial undeformed state. iv) Inlet flow rate insensitivity: The fast volume output is not sensitive to the flow rate at the valve inlet, as the output is generated from the air transfer between the constituent shells during snapping. In the following sections, we first study the snapping and interaction between the elastic shells of our bi‐shell valve to understand the role that each of them plays during deflation. We then characterize the response of each shell within a wide geometric space and map the range of fast volume outputs our bi‐shell valve can achieve. Finally, we demonstrate the application of our bi‐shell valve to thrust the motion of an object along a guided track.

## 2 Results and Discussion

### 2.1 The Bi‐Shell Valve Concept: Tapping into Shell Snapping Interaction

**Figure 1:**
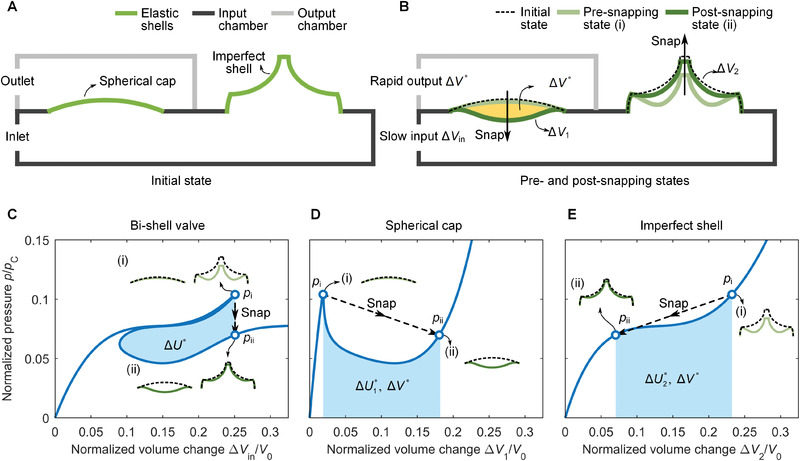
Bi‐shell valve with snap‐through response under volume control. A) Schematic of the bi‐shell system in its unloaded state; the valve comprises two thin elastomeric shells, one with perfect and the other with imperfect spherical geometry, connected through an input chamber (below both) and output chamber (above the spherical cap). B) Pre and post snapping states of the bi‐shell valve under incremental deflation Δ*V*_in_. Prior to instability (light green) the spherical cap is almost locked in its initial shape yielding no change in volume Δ*V*_1_ as opposed to its imperfect counterpart, which can deform downward smoothly without snapping from the initial state to state (i) and store elastic energy due to its compliance with an accrued change in volume Δ*V*_2_. There is no snapping during these stages because the instability is triggered by the buckling of the spherical cap. Upon snapping (dark green), the former bounces down to its buckled shape with a volume change of ΔV*, whereas the latter springs up from state (i) to state (ii) with a release of ΔV*. In yellow is the resulting ΔV*, i.e., the volume difference between pre (i) and post (ii) snapping states, which describes the rapid change in volume generated by the snapping of the spherical cap. C–E) Pressure‐volume responses of the entire bi‐shell system (C), the spherical cap solely (D), and the imperfect shell only (E). The normalization factors of pressure and volume are the buckling pressure $P_{C}=2E{\left(t/R\right)}^{2}/\sqrt{3(1-\nu^{2})}$ and enclosed volume *V*_0_ = 2*πR* ^3^/3 of a baseline hemispherical shell with radius identical to the radius *R* on the bottom plane of the constituent shells.

Our bi‐shell valve consists of two interacting elastic shells that cooperate upon snapping to generate a rapid change in volume in response to a slow volume input. **Figure** **1**A shows the bi‐shell valve concept in its undeformed state. Beneath the shells, an input chamber connecting the shell inner volumes provides deflation under volume control as well as pressure control, if required. On the left‐hand side is a perfect spherical cap which exhibits an unstable response, typical of elastic thin shells with a high peak of pressure attained in the nearly undeformed state, followed in turn by a rapid fall of pressure into a plateau leading to full eversion.^[^ ^46^ ^]^ On the right‐hand side is a hemispherical shell featuring a large axisymmetric imperfection in the form of an elliptical arc traced away from the pole. We select this imperfect shell for its stable response over a large change in volume that can be effectively tuned by the geometry of the imperfection, thus attaining a capacity to provide increasing pressure resistance.^[^ ^47^ ^]^ The bi‐shell valve operates through a slow deflation (negative pressure) imparted by the inlet of the input chamber and delivers a fast deflation via the outlet at the output chamber. Since the pressure of the bi‐shell valve remains negative during the operation, we have made here the assumption of neglecting the negative sign for the convenience of the analysis.

By combining the two shell architectures, a perfect spherical cap and an imperfect shell, each with its own distinct response, into one bi‐shell system (Figure 1A), we can program the mechanism of deformation, impart a desired sequence of deflation, and code the global performance of our valve. Our goal is to capitalize on shell snapping interaction to generate a function that adds to the control‐flow function of existing soft valves that would be otherwise unattainable through current concepts involving either snapping of a single spherical cap or other strategies.

We start with a description of the qualitative response of the system deformation. Figure 1B illustrates that a slow deflation of the input chamber through the inlet (Δ*V*_in_), brings each shell into different states. At the onset of deflation (light green), the pole of the imperfect shell deforms downward smoothly without snapping from the initial state to state (i), whereas the perfect cap barely undergoes any deformation, hence retaining its initial state (hidden line overlaid on light‐green solid line). There is no snapping during these stages because snapping is triggered by the buckling of the spherical cap. After snapping (dark green), the imperfect shell springs back from state (i) to state (ii), whereas the spherical cap everts downward from its initial upward position.

To capture the snapping behavior in quantitative terms, we determine the pressure‐volume response of the bi‐shell valve. We first assess how the volume change of the input chamber governs the pressure of the input chamber, and then normalize these values respectively by the buckling pressure $P_{C}=2E{\left(t/R\right)}^{2}/\sqrt{3(1-\nu^{2})}$ and the enclosed volume *V*_0_ = 2*πR* ^3^/3 of a baseline hemispherical shell with radius identical to the radius *R* on the bottom plane of the constituent shell. These metrics are the characteristic axes of the plot reported in Figure 1C. While deflation can be expected to generate negative values of both volume and pressure, here we consider their signs as positive for the convenience of the analysis. The portion of the curve up to (i) describes the initial deformation prior to snapping when the pressure hits the snapping pressure *p*_i_. A further deflation triggers a snap‐through instability with a configurational change in both shells, now reaching simultaneously their second state (ii) of equilibrium. The process is characterized by a drop of pressure to *p*_ii_ with the spherical cap eversion synchronous to the snap back of the imperfect shell. Since the outer side of the former shell is enclosed by the output chamber, its downward snapping produces a rapid change in volume (yellow area ΔV*, Figure 1B), which is bounded by the pre‐ and post‐snapping states of the spherical cap in the output chamber. The hallmark of this concept is that the release of volume to slow deflation is fast and would be otherwise inaccessible by employing either shell individually, as shown later. As per the energy and volume change in the input chamber, the snapping event causes a release of the previously stored elastic energy in both shells (ΔU*) but with almost no change in volume (less than 0.54% of the total volume); this outcome is due to the extremely small difference in pressure (less than 550 Pa) between the input chamber and the atmosphere (see the Supporting Information for details).

### 2.2 Inferring the Bi‐Shell Valve Behavior from the Individual Response of Each Shell

As described above, the deformation of our bi‐shell system (Figure 1C) is the result of the collective response of the constituent shells, each cooperating distinctly during deflation and snapping. To understand the interaction between them and each of their roles, here we examine each shell separately, investigate their individual responses when deflated separately, and correlate them to the system behavior.

Figure 1D pertains to the spherical cap on its own, and Figure 1E to the imperfect shell on its own. The former (blue path in Figure 1D) shows the characteristic highly unstable response of a perfect elastic shell. A small volume change (Δ*V*_1_/*V*_0_ < 0.02) makes the pressure quickly escalate to a high critical value (*p*/*p*_C_ = 0.104, *p*_i_), which immediately drops to a lower plateau (*p*/*p*_C_ = 0.05), spanning a wide range of volume change (0.05 < Δ*V*_1_/*V*_0_ < 0.2). After the plateau, the pressure increases rapidly again. There is no limit point in volume because the spherical cap selected here is thick and shallow, similar to the findings of Gorissen et al.^[^ ^31^ ^]^; the elements that are here important to trigger snapping are the initial peak in pressure, the subsequent softening, and a final increase in pressure as noted by Overvelde et al.^[^ ^32^ ^]^. The latter shell of our valve exhibits a stable pressure response (blue path in Figure 1E) over an increasing volume change Δ*V*_2_, and its buckling mode is enabled by the size and location of the imperfection (see the Supporting Information for details). The pressure‐volume path (blues) has three portions: an initial rapid increase of pressure for small values of the volume change (Δ*V*_2_/*V*_0_ < 0.1), followed by a stable plateau offering a gradual increase of pressure for intermediate values of Δ*V*_2_/*V*_0_, and finally a steep increase of pressure. While the blue paths in Figure 1C,D,E represent the static equilibrium responses of each system, the black hidden ones with arrow mark the direction from the pre to the post snapping state.

Once the individual shells join through the input chamber, a concerted deformation (Figure 1C) takes place to ensure equilibrium of volume and pressure. Equilibrium requires the balance of their pressure in the stable post‐snapping state (see the Supporting Information). The conservation of volume requires the input volume change Δ*V*_in_ to equal the sum of the volume changes of the individual shells, Δ*V*_1_ and Δ*V*_2_.^[^ ^32^, ^33^, ^48^, ^49^ ^]^ The onset of snapping is mainly controlled by the spherical cap, which upon winning the critical pressure *p*_i_, cannot accommodate any further increase in pressure, resulting in a sudden drop of pressure due to its elastic instability (Figure 1D). It is this event that triggers the snapping of both shells (black paths in Figure 1C,D,E). Each shell springs into its own snapped state in a swift manner. During snapping, the spherical cap collapses due to its reduced pressure resistance and propels the air in the input chamber toward the adjacent shell for an upward push; the sudden deflation of the former inflates the latter.

As with other forms of elastic deformation, during snapping each shell can store and release elastic energy (cyan areas in Figure 1), and their amounts correlate with those of the entire system. In particular, the deflation of the spherical cap by a volume change ΔV* is accompanied by the storage of elastic energy $\Delta U_{1}^{*}$ (Figure 1D), while the inflation of the imperfect shell by a volume of ΔV* corresponds to the elastic energy release $\Delta U_{2}^{*}$ (Figure 1E). The difference between them, $\Delta U^{*}=\Delta U_{2}^{*}-\Delta U_{1}^{*}$, is the elastic energy released by the bi‐shell valve due to the snapping of both constituents (Figure 1C), a notion consistent with the existing literature.^[^ ^31^, ^32^, ^33^, ^48^, ^49^ ^]^ In addition, we note that the energy stored by both shells in the linear portion of the pressure‐volume curves is not considered here because it does not contribute to the energy output during snapping.

### 2.3 Experimental Investigation

**Figure 2:**
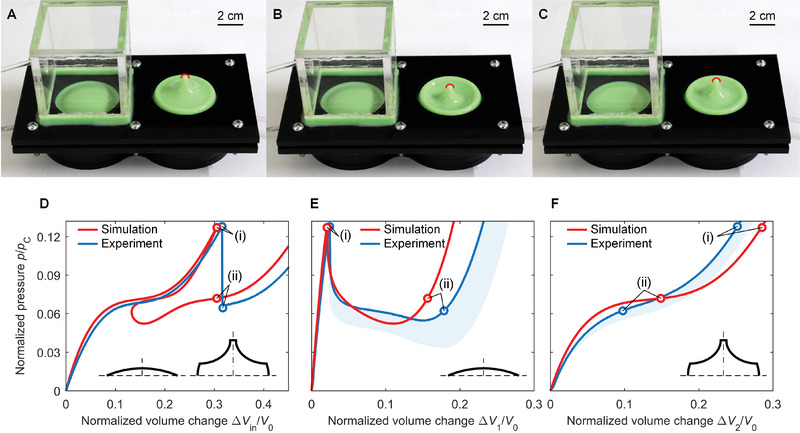
Experiment of the bi‐shell valve. Photographs of bi‐shell valve: A) initial state, B) state (i) before snapping, and C) state (ii) after snapping. The top of the imperfect shell is outlined with a red cap to emphasize the difference between states. D–F) Pressure‐volume responses of the input chamber, the spherical cap, and the imperfect shell. *P*_C_ is the theoretical critical pressure of a perfect spherical shell and *V*_0_ is the volume of a hemisphere. The cyan domains in (E) and (F) show the envelope of the experimental response of three tested samples.

**Figure** **2**A shows our bi‐shell valve fabricated with two thin shells (green) made out of Zhermack Elite Double 32 (Zhermack, Italy), along with acrylic input (painted in black) and output (transparent) chambers (see the Supporting Information for details). Figure 2B,C illustrates the mechanism of deformation at two sequential instants (pre and post snapping) upon deflation, with configurations that parallel those shown in Figure 1. In the imperfect shell, an axisymmetric mode of deformation first accrues until state (i) is reached, while the spherical cap holds its undeformed shape under initial pressure reduction (Figure 2B). Further deflation causes both shells to snap into distinct states (ii), the imperfect shell nearly springing upward to its initial configuration and the perfect shell buckling downward (Figure 2C and Movie S1; Supporting Information).

Figure 2D–F shows the pressure‐volume change curves obtained from both our analyses and experiments (see the Supporting Information for details). The responses on all fronts, i.e., bi‐shell valve, spherical cap, and imperfect shell, show a snapping behavior matching those in Figure 1C–E. In Figure 2D, the experimental pressure of the input chamber (blue curve) first increases smoothly upon air deflation until state (i) (Δ*V*_in_/*V*_0_ = 0.32), and then plunges from *p*_i_/*p*_C_ = 0.128 to *p*_ii_/*p*_C_ = 0.064. Here, the experimental results confirm that the drop in pressure is triggered by the snapping of the spherical cap, which collapses downward (Figure 2C). The prediction of our numerical simulation (red curve) agrees with the experimental results, with the predicted pre‐ and post‐snapping states (i) and (ii) resembling those of their experimental counterparts as quantified below.

While the volume change and energy output of the bi‐shell valve cannot be directly calculated from the experimental curve (pressure‐volume change) in Figure 2D, we can still estimate their values through the separate response of their elastic constituents, i.e., spherical cap and imperfect shell (blue curves in Figure 2E,F). The post‐snapping pressure is determined through the equilibrium condition the two shells should satisfy at *p*_ii_/*p*_C_ = 0.062 (Figure 2E,F), which is close to the value, *p*_ii_/*p*_C_ = 0.064, from our experimental result in Figure 2D. The relative error between the estimated and measured post‐snapping pressure is below 4%, demonstrating the accuracy of our estimation. From state (i) to state (ii), the volume change due to snapping is then obtained from the separate response of each shell as ΔV*/*V*_0_ = 0.154, while the energy output is ΔU*/(*p*_C_ *V*_0_) = 3.57 × 10^−3^. Our analysis provides an accurate prediction of the valve response, described by a volume change of ΔV*/*V*_0_ = 0.135 and a released energy of ΔU*/(*p*_C_ *V*_0_) = 3.63 × 10^−3^. To minimize the error between numerical and experimental results, we employ a realistic model that accounts for any non‐uniformity of shell thickness caused by manufacturing as well as for the initial deformation due to clamping (see the Supporting Information for details). The discrepancy, especially at larger strains, is attributed to imperfections in shell geometry that emerge during fabrication. This might include geometric deviation of the mold from the nominal design, as well as defects in the base material, such as microvoids.

The results from both analyses and experiments (Figures 1 and 2) reveal that shell interaction governs the bi‐shell valve behavior, which in turn can be retrieved by combination of the individual shell response. These insights not only enable us to understand the role of each shell during snapping, but also provide principles for valve design involving multiple shells. We propose a two‐steps approach (see Figure S16 for a flowchart of the design process in the Supporting Information), where the bi‐shell performance is defined by 4 metrics: the upper bounds of volume and energy, $\Delta V_{\mathrm{upper}}^{*}$ and $\Delta U_{\mathrm{upper}}^{*}$, as well as the working ranges of ΔV* and ΔU*, i.e., the variation of volume change and released energy within their respective bounds. The first step involves examining the spherical cap only, which sets the valve performance limits, and aims at determining the upper bounds ($\Delta V_{\mathrm{upper}}^{*}$ and $\Delta U_{\mathrm{upper}}^{*}$) of the valve output. These bounds set the upper boundaries for the bi‐shell valve performance, i.e., the bi‐shell system with a given spherical cap cannot exceed them for any geometric scenario of the imperfect shell. In the second step, we determine the attainable ranges of volume change ΔV* and released energy ΔU* for the bi‐shell by exploring the design space of the imperfect shell for a prescribed geometry of the spherical cap. With this approach, we can ensure that we reach the full potential of both shells, thereby yielding a valve output close to the achievable maximum.

### 2.4 Upper Bounds of Volume Change and Released Energy

**Figure 3:**
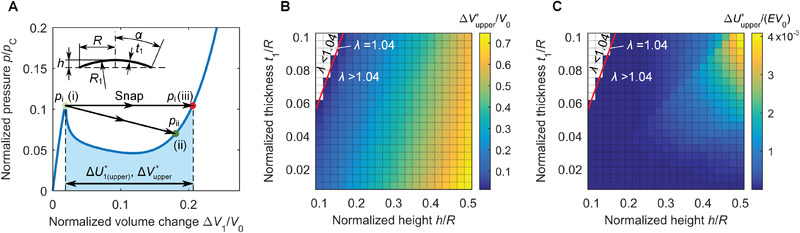
Upper bounds of performance for perfect shell limiting our bi‐shell valve output. A) Representative pressure‐volume curve of a perfect spherical shell. B) Upper bound of volume change, $\Delta V_{\mathrm{upper}}^{*}/V_{0}$, plotted as a function of the normalized thickness *t*_1_/*R* and normalized height *h*/*R*. C) Upper bound of released energy of the bi‐shell valve, $\Delta U_{\mathrm{upper}}^{*}/\left(EV_{0}\right)$, plotted as a function of *t*_1_/*R* and *h*/*R*. *λ* = (12(1 − *ν* ^2^))^1/4^(*R*_1_/*t*_1_)^1/2^ *α* with *ν* the Poisson's ratio. Normalization factors of the volume change and released energy are *V*_0_, the volume of a hemisphere with radius *R*, and, Young's modulus *E*.

The goal is to find the first two performance metrics of our bi‐shell valve, i.e., the maximum values of volume change, $\Delta V_{\mathrm{upper}}^{*}$, and released energy, $\Delta U_{\mathrm{upper}}^{*}$, our bi‐shell valve can attain. As described earlier, the spherical cap sets the upper performance limit of our bi‐shell valve, and is studied here as standalone shell. The modified Riks method is employed to simulate the deflation of a spherical cap subject to uniform pressure and parametrically map $\Delta V_{\mathrm{upper}}^{*}$ and $\Delta U_{\mathrm{upper}}^{*}$ upon snapping. The parameters defining the spherical cap geometry (inset in **Figure** **3**) are the radius in the base plane (*R* = 25mm), the normalized thickness (varying between *t*_1_/*R* = 0.01, and 0.1), and the normalized height (ranging from *h*/*R* = 0.1 to 0.5).

Figure 3A shows a representative curve of pressure versus volume change for a perfect spherical cap. The end point (light green) of the pre‐snapping path denotes the pressure peak *p*_i_ (state (i)), which is typically above the end point of the post‐snapping path (dark green designating state (ii)) at *p*_ii_. As shown in Figure 1C–E, the drop of pressure from *p*_i_ to *p*_ii_ represents the reduction in pressure resistance offered by the imperfect shell. The position of state (ii) depends on shell interaction, and can be at any point along the post‐snapping curve in Figure 3A, even at sites approaching state (iii). If snapping brings state (ii) to coincide with state (iii) (red point), then the volume change and the stored energy of the spherical cap reach the maximum values, $\Delta V_{\mathrm{upper}}^{*}$ and $\Delta U_{1(\mathrm{upper})}^{*}$ (Figure 3A), which are the upper bounds illustrated in Figure 3A respectively in the lower part and in cyan. At this state, the energy released by the bi‐shell valve, $\Delta U_{\mathrm{upper}}^{*}=p_{i}\Delta V_{\mathrm{upper}}^{*}-\Delta U_{1(\mathrm{upper})}^{*}$, also reaches the upper bound, (see the Supporting Information for details). $\Delta V_{\mathrm{upper}}^{*}$ and $\Delta U_{\mathrm{upper}}^{*}$ describe an ideal scenario for bi‐shell valve where the pressure of the imperfect shell is insensitive to snapping, i.e., no pressure drop is developed during snapping and no change in pressure takes place (see the Supporting Information for details). In the following, we show that the upper bounds obtained for the perfect spherical cap can be used to characterize the output performance of our bi‐shell valve as a function of the cap geometry.

To assess the snapping performance with respect to changes in shell geometry, we first define a widely used parameter of the perfect cap shape, which incorporates the dimensionless size and thickness of the spherical cap. This is *λ* = (12(1 − *ν* ^2^))^1/4^(*R*_1_/*t*_1_)^1/2^ *α*,^[^ ^50^, ^51^ ^]^ where *R*_1_ is the radius of the spherical cap, *t*_1_ is the thickness, *α* (Figure 3A) is the edge angle measured from the axis of symmetry, and *ν* the Poisson ratio. *λ* enables for the discrimination of areas of the design space, normalized thickness *t*_1_/*R* versus normalized height *h*/*R*, with snap‐through instability from those without. This is illustrated (Figure 3B,C) for both upper bounds of volume change and released energy. For *λ* < 1.04 (white area in the upper left corner), the spherical cap is thick and shallow, and no snap‐through takes place; here there is only shell deflation with a smooth increase of pressure that cannot generate any rapid volume change. In contrast for *λ* > 1.04, the shell, thinner and deeper in geometry, undergoes snap‐through for all combinations of *t*_1_/*R* and *h*/*R*. As per the values of the upper bounds for volume change and released energy, the results in Figure 3B,C help to gain insights into the geometric parameters that govern the output performance of the bi‐shell valve. From their contour plots, we observe that $\Delta V_{\mathrm{upper}}^{*}$ increases monotonically with the normalized height *h*/*R* and its span is sizeable, from $\Delta V_{\mathrm{upper}}^{*}/V_{0}=0$ to 0.75 (Figure 3B). The main implications is that a shell with higher *h*/*R* in its initial state, inherently encloses a larger inner volume, thus outlining a geometry capable of generating large change in volume upon snapping to the fully everted state. On the other hand, compared to *h*/*R*, the normalized thickness *t*_1_/*R* exerts a minor influence on the volume change. As per the energy release, Figure 3C shows that $\Delta U_{\mathrm{upper}}^{*}$ increases from $\Delta U_{\mathrm{upper}}^{*}/\left(EV_{0}\right)=0$ to 0.004 with both *h*/*R* and *t*_1_/*R*. In comparison, shells with larger *h*/*R* can generate more ample change in volume during snapping, with a larger *t*_1_/*R* providing a higher pressure. The maximum released energy $\Delta U_{\mathrm{upper}}^{*}/\left(EV_{0}\right)$ is obtained at the upper right corner of Figure 3C, where both *h*/*R* and *t*_1_/*R* take their largest values, and both volume and pressure changes have large values.

The results in Figure 3B,C become useful for soft robotic applications. $\Delta V_{\mathrm{upper}}^{*}$ and $\Delta U_{\mathrm{upper}}^{*}$ set performance limits that apply to our bi‐shell valve. *h*/*R* and *t*_1_/*R* are the governing dimensionless parameters. Through the proper combination of their *h*/*R* and *t*_1_/*R*, we can program the max volume change and released energy of the valve from the geometry of the cap only. For example, a sufficiently large *h*/*R* is needed to generate enough volume change and energy for soft actuators, whereas a small *h*/*R* can limit the upper bound of volume change $\Delta V_{\mathrm{upper}}^{*}$ and released energy $\Delta U_{\mathrm{upper}}^{*}$ to within a safety threshold, e.g., to prevent accident in human‐robot interaction.^[^ ^52^ ^]^ On the other hand, the normalized thickness *t*_1_/*R* has low to mild influence on the volume change $\Delta V_{\mathrm{upper}}^{*}$, but strong on the upper bound of the released energy $\Delta U_{\mathrm{upper}}^{*}$.

### 2.5 Bi‐Shell Valve Performance in Service: Attainable Values of Volume Change and Released Energy

**Figure 4:**
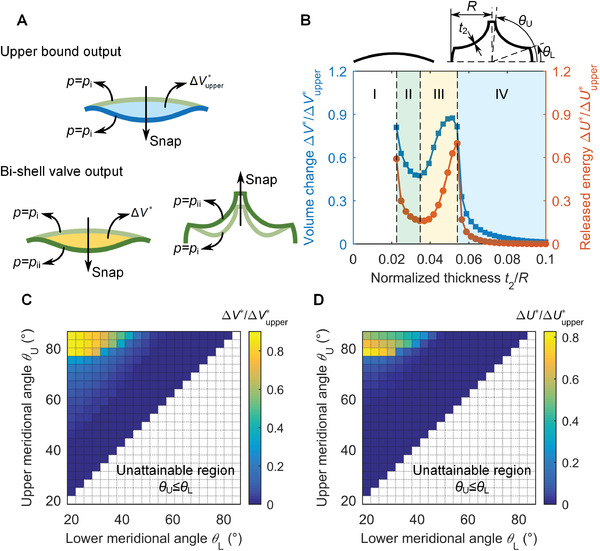
Bi‐shell valve output. A) Upper bound output at *p* = *p*_i_ and bi‐shell valve output at *p* = *p*_ii_. B) Performance output of bi‐shell valve as a function of the normalized thickness *t*_2_/*R* of the imperfect shell for given geometry of the spherical cap. C) Volume change, $\Delta V^{*}/\Delta V_{\mathrm{upper}}^{*}$, as a function of the meridional angles defining the upper and lower boundary of imperfection, *θ*_U_ and *θ*_L_. D) Released energy, $\Delta U^{*}/\Delta U_{\mathrm{upper}}^{*}$, as a function of the meridional angles defining the upper and lower boundary of imperfection, *θ*_U_ and *θ*_L_. Normalization factors of the volume change and released energy are the upper bounds $\Delta V_{\mathrm{upper}}^{*}$ and $\Delta U_{\mathrm{upper}}^{*}$. The unattainable region in white corresponds to cases where *θ*_U_ is below or equal to *θ*_L_.

While the cap snapping bounds the theoretical output of the bi‐shell valve $\Delta V_{\mathrm{upper}}^{*}$ and $\Delta U_{\mathrm{upper}}^{*}$(top sketch in **Figure** **4**A), it is the interaction between the two shells that governs the values of volume change ΔV* and energy ΔU* that the bi‐shell system can actually release during operation (low sketch in Figure 4A). Our goal here is to find this second set of metrics (ΔV* and ΔU*) defining our bi‐shell valve performance. We do so by first prescribing the geometry of the spherical cap (*t*_1_/*R* = 0.05, *h*/*R* = 0.2, and *R* = 25mm), and then systematically exploring the design space of the imperfect shell. This step entails finding the range of ΔV* and ΔU* that can be obtained by varying the normalized thickness, *t*_2_/*R*, within a representative span (0.02–0.1), and the meridional angles of the upper and lower boundaries, *θ*_U_ and *θ*_L_, from 20° to 85°, a demonstrative range that we select here for these angles.

Figure 4B shows the attainable volume change $\Delta V^{*}/\Delta V_{\mathrm{upper}}^{*}$ (blue plot) and released energy $\Delta U^{*}/\Delta U_{\mathrm{upper}}^{*}$ (orange plot) as a function of *t*_2_/*R*, the normalized thickness of the imperfect shell, for given meridional angles (*θ*_U_ = 85° and *θ*_L_ = 20°). Here we can identify four regimes, each yielding a distinct performance of the bi‐shell valve (see the Supporting Information S5 for the analysis of the buckling modes). For *t*_2_/*R* ≤ 0.02 (regime I), no snapping occurs because the imperfect shell is much more compliant than the spherical cap and collapse brings it to full eversion before the pressure is able to reach the snapping pressure of the spherical cap. For 0.02 < *t*_2_/*R* < 0.035 (regime II), the volume change ΔV* decreases with *t*_2_/*R* from 81% to 47% of the upper bound $\Delta V_{\mathrm{upper}}^{*}$, and the released energy Δ*U* drops from 59% to 16% of the upper bound $\Delta U_{\mathrm{upper}}^{*}$. Within this range, the minimum values of Δ*V* and Δ*U* are low compared to the upper bounds generated by the spherical cap; this implies that the imperfect shell can only trigger a small portion of $\Delta V_{\mathrm{upper}}^{*}$and $\Delta U_{\mathrm{upper}}^{*}$. On the other hand, in regime III (0.035 < *t*_2_/*R* < 0.055), both the volume change and released energy of the bi‐shell valve increase rapidly with *t*_2_/*R*. The volume change ranges from $\Delta V^{*}/\Delta V_{\mathrm{upper}}^{*}=0.49$ to 0.87 and the released energy ($\Delta U^{*}/\Delta U_{\mathrm{upper}}^{*}$) spans the range 0.16 – 0.70. A further increase in *t*_2_/*R* (regime IV) leads to an abrupt drop in both volume change and released energy, followed by a plateau that gradually approaches the value of zero. For these shells, snapping offers very modest volume change and released energy.

If the meridional angles, *θ*_U_ and *θ*_L_, are both considered as design parameters, then a larger design space emerges for both $\Delta V^{*}/\Delta V_{\mathrm{upper}}^{*}$and $\Delta U^{*}/\Delta U_{\mathrm{upper}}^{*}$. This is shown respectively in Figure 4C,D. Here the normalized thickness is prescribed to the value *t*_2_/*R* = 0.05 to ensure that $\Delta V^{*}/\Delta V_{\mathrm{upper}}^{*}$ and $\Delta U^{*}/\Delta U_{\mathrm{upper}}^{*}$ can take the largest output depicted in Figure 4B. In terms of volume change $\Delta V^{*}/\Delta V_{\mathrm{upper}}^{*}$, the yellow upper left corner in Figure 4C indicates large values, whereas the other regions in blue show a modest volume change $\Delta V^{*}/\Delta V_{\mathrm{upper}}^{*}$ almost close to zero. For *θ*_L_ ≤ 33°, we find that the volume change $\Delta V^{*}/\Delta V_{\mathrm{upper}}^{*}$ first increases slowly with the upper meridional angle *θ*_U_ until an abrupt increase from $\Delta V^{*}/\Delta V_{\mathrm{upper}}^{*}=0.3$ to 0.9 appears at *θ*_U_ = 78.5°. With further increase in *θ*_U_, the volume change $\Delta V^{*}/\Delta V_{\mathrm{upper}}^{*}$ stays almost constant for the plotted range *θ*_U_ ≤ 85°. As per the released energy, Figure 4D shows a contour plot similar to that of the volume change (Figure 4C). For *θ*_L_ ≤ 33°, an abrupt increase of released energy from $\Delta U^{*}/\Delta U_{\mathrm{upper}}^{*}=0.15$ to 0.83, the maximum, appears at *θ*_U_ = 78.5°. A further increase in *θ*_U_, however, yields reduced values of the released energy, as opposed to $\Delta V^{*}/\Delta V_{\mathrm{upper}}^{*}$ which remains almost constant for *θ*_U_ ≥ 78.5°. As with Figure 4B, the maximum values of the volume change ΔV* and released energy ΔU* in Figure 4C,D can only be attained in a narrow design space (yellow) of the imperfect shell. This zone is key to maximize the valve output, i.e., to release a large amount of energy. Its extent is governed by the interaction between the spherical cap and the imperfect shell. In particular, this yellow zone describes bi‐shell valves in which the plateau pressure of the imperfect shell is located between the pre and post‐snapping pressure *p*_i_ and *p*_ii_ of the spherical cap (see the Supporting Information for further details).

In summary, the upper bounds ($\Delta V_{\mathrm{upper}}^{*}$ and $\Delta U_{\mathrm{upper}}^{*}$) in Figure 3 and the valve outputs (ΔV* and ΔU*) in Figure 4 provide guidelines of practical use for the design of our bi‐shell valve. First, the upper bounds in Figure 3 can guide the selection of the spherical cap that has the potential to generate a proper valve output. Second, for a given spherical cap, Figure 4 (along with Figure S13, Supporting Information) summarizes the range of valve output that can be tuned with the geometry of the imperfect shell as well as identifies distinct regimes of buckling modes. In this case, despite the size of the design space, only a small window is available for the imperfect shell (yellow area in the upper left corner of Figure 4C,D) to generate a valve output that is close to the upper bound. The insights here gained point out that *θ*_U_ = 85.9° and *θ*_L_ = 20° are among the best geometric parameters of the imperfect shell that can elicit the large volume change that we observe in our experiments (Figure 2 and Movie S1, Supporting Information). While the results in Figure 4 mapping the geometric role of the imperfect shell are for a specific geometry of the spherical cap, our analysis can be straightforwardly applied to spherical caps with other geometries; this merely requires updating the spherical cap geometry and replot the bi‐shell valve performance of Figure 4.

### 2.6 Application of the Bi‐Shell Valve

We now capitalize on the performance assessment and physical insights gained so far to demonstrate the capacity of our bi‐valve to leverage shell snapping interaction for rapid actuation of fluidic soft actuators. As illustrative application, we employ a soft pneumatic striker that we actuate through our bi‐shell valve. The goal is to make the striker suddenly move in response to the concerted snapping of the valve shells, deflated merely with slow volume input, propelling a table tennis ball along a guided track.

**Figure 5:**
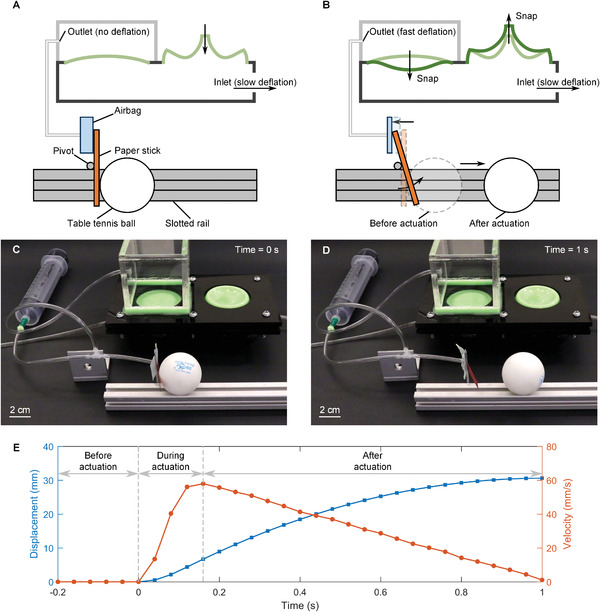
A striker rapidly actuated by bi‐shell valve to propel motion of table tennis ball. A) The striker consists of an airbag and a paper stick. The airbag is connected to the bi‐shell valve. The paper stick is constrained by a pivot fixed to the slotted rail to enable swinging around its midpoint. A table tennis ball placed on the slotted rail is in contact with the paper stick. B) Upon snapping of the bi‐shell valve, the airbag rapidly deflates to swing the paper stick, which rotates anti‐clockwise to hit the ball along the rail. C,D) Photographs prior and post the striker hit (Movie S2, Supporting Information). A syringe is used to inflate the airbag from its natural flat condition to its fully inflated state; it ceases to provide any increase of air volume once full inflation is reached. E) Displacement and velocity of the table tennis ball as a function of time. Time = 0 s is the instant when the striker is actuated by the bi‐shell valve.

**Figure** **5**A illustrates the schematic of our "soft punch" system. It consists of an airbag that contracts upon deflation and a paper stick that is driven by the airbag to hit and drive the table tennis ball along a slotted rail. Below the airbag, a pivot is fixed to the slotted rail at its midpoint, around which the paper stick can swing freely through the air. The system works as follows. First, the bi‐shell valve is deflated to a state near to its pre‐snapping state; during this process, the elastic strain energy is stored in the shells interacting through their input chamber. Second, the airbag is connected to the output chamber. Third, further deflation is applied at a low flow rate; this enables the pressure in the input chamber underneath the shells to reach the buckling load of the spherical cap. At this stage, the spherical cap snaps downward and swiftly engages its imperfect counterpart to snap upward. Shell cooperation is now capitalized. The elastic energy and volume change hoarded in the imperfect shell before snapping is now released to propel fast deflation of the spherical cap. Since the output chamber surrounding the spherical cap connects to the airbag, the accrued rapid volume change is dispensed to fast deflate the airbag (Figure 5B). Since the tubing connecting the valve and soft actuator is short, the flow resistance or backpressure of the tubing is negligible. As a result, the upper part of the stick retracts and swings backward around the pivot, whereas its lower portion knocks the ball forward, away from its initial position.

Figure 5C,D shows the physical realization of the schematics shown in Figure 5A,B. In Figure 5C, the airbag is connected through a tube to the output chamber of our bi‐shell valve (Figure 5A), while a syringe inflates the airbag from its natural flat condition to its fully inflated state. Once full inflation is reached, the syringe ceases to provide any increase of air volume. Deflation of the input chamber at the inlet is at a constant flow rate of 3 mL min^−1^. After snapping, the airbag deflates almost instantly until air exhaustion, a condition where the air bag becomes fully rigid. The absence of air in the airbag impedes the spherical cap to reach full eversion, rather only a dimple forms on its top (Figure 5D). Despite full eversion is not being attained, the partial snapping enables the striker to hit the table tennis ball up to 3 cm from its initial position (Movie S2, Supporting Information). We emphasize that if the bi‐shell valve cannot generate sufficient volume change to meet the requirements of a given soft robotic application, e.g., when the stiffness of the actuator influences the bi‐shell valve output, then the valve design (see Figure S16 for design flow chart in the Supporting Information) can be readjusted through the metrics introduced in the maps of Figures 3 and 4. This shows a versatile design for our valve capable of matching the actuator performance, and reaching the target volume change regardless of the actuator performance.

Figure 5E shows the displacement and velocity imparted by the striker to the table tennis ball. Before actuation, the ball rests in its initial position with both displacement and velocity equal zero. This reflects the condition of the airbag being not deflated. Upon snapping, the ball quickly accelerates to reach a speed of 58 mm s^−1^ within 0.16 s. After actuation, the ball keeps moving on the rail until the speed gradually declines to zero, due to the combined effects of rail friction, rail unevenness and air drag.

**Figure 6:**
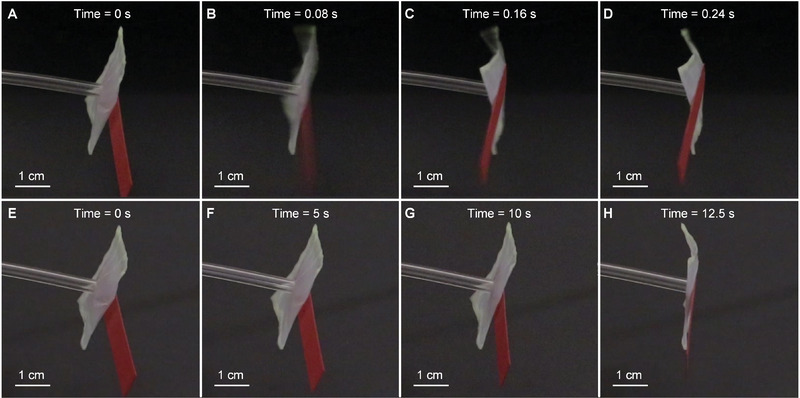
A–D) Fast actuation of the striker enabled by the bi‐shell valve. The striker can reach full deflation in 0.16 s and reach a state with no intrinsic vibration at 0.24 s. E–H) Slow actuation of the striker due to the absence of the bi‐shell valve. More than 10 s are required for actuation. For both tests, the actuation time is counted from the 0 s instant.

To further prove that the fast movement of the striker is enabled by our bi‐shell valve, we perform two additional tests that compare the striker actuation in two scenarios (**Figure** **6**): one with the bi‐shell valve and the other without the valve. In this set of tests, we focus on the response of the striker only, and hence we remove the table tennis ball and the rail. For the first test (Figure 6A–D), we connect the striker to the bi‐shell valve and slowly deflate the valve at a constant flow rate of 3 mL min^−1^. The airbag quickly deflates within 0.16 s upon snapping (Movie S3, Supporting Information), a result in agreement with the time required by the table tennis ball to accelerate. For the second test (Figure 6E–H), we remove the bi‐shell valve and directly deflate the airbag at the identical flow rate (3 mL min^−1^). What we observe here is that the airbag slowly shrinks to the fully deflated state over about 13 s (Movie S4, Supporting Information), i.e., roughly 80 times slower than the first scenario. The comparison of these test results attests that our bi‐shell valve is responsible for the fast movement of the striker. In summary, the experiments above demonstrate that our bi‐shell valve can achieve fast actuation through a slow volume input. While existing snapping valves^[^ ^22^, ^29^, ^43^, ^44^, ^45^ ^]^ can provide binary control of a given flow, our concept leverages shell cooperation and snapping interaction to quickly impart a fast volume change that can be used for actuation.

### 2.7 Discussion

Widely used methods currently available for rapid actuation mainly resort to pressure‐controlled strategies that require a bulky system of pressure supply, sensors, hard valves, and control algorithms.^[^ ^4^, ^15^ ^]^ Other methods to achieve rapid actuation either employ explosive chemical reaction^[^ ^39^, ^40^, ^41^, ^42^ ^]^ or exploit a structural instability embedded within the actuator, which would require the integration of snapping spherical caps^[^ ^31^ ^]^ or balloons^[^ ^32^, ^36^, ^37^ ^]^ into the architecture of the actuator. In contrast, our bi‐shell valve does not rely on the fast volume input from pressure control or any modifications to the actuator design. It can be easily implemented with a simple volume input dispensed through a syringe and connected to an existing actuator. We note that the pressure output is governed by the constituent shells. In this work, the snapping pressure of the bi‐shell valve is in the range 405–549 Pa (Figure S6, Supporting Information). Yet, the snapping pressure can be increased by stiffening the shells. On this front, two strategies can be followed. One is to thicken the shell and scale up the snapping pressure to a value as large as 10 kPa, as demonstrated in the literature.^[^ ^29^ ^]^ Another way is to choose a material with Young's modulus higher than that of our prototype valve (see the Supporting Information for details). Both methods can be used either individually or in combination to further amplify the pressure output and meet the design requirements imposed by the application.

In addition, our valve performance could not be achieved by employing one single shell, as in existing valve designs delivering mainly fluid control function.^[^ ^22^, ^29^, ^43^, ^44^, ^45^ ^]^ The reason is that the flow rate difference between the valve input and the valve output is enabled by the volume rapidly transferred between the constituent shells during snapping. This allows to convert the low flow rate at the inlet to a fast flow rate at the outlet. If only one shell were used, either the spherical cap or the imperfect shell, no fast actuation would be attainable because there would be no means in place for flow‐rate conversion, i.e., no fast transfer of air volume can be accomplished. Another advantage is the self‐adaptivity of its volume output to that of the actuator. Our experiments have shown that the valve can be autonomously adjusted to yield a volume output that is compatible with the volume of the actuator at the outlet, hence preventing the actuator from any possible damage caused by excessive deflation. Furthermore, the hyperelastic constitutive law of the shell base‐material guarantees reversible and repetitive snapping imposed by a cyclic loop of deflation and inflation (Movie S5, Supporting Information), which is common for soft robots and actuators.^[^ ^29^, ^31^ ^]^ In our demonstration, the choice to show the bi‐shell valve on a system containing rigid components is only for the convenience to observe the snapping event and the interaction of the constituent shells. To integrate our valve within a soft robot, the output chamber can be merged with the interior of the soft actuator, while the whole system of the bi‐shell valve and the soft actuator can be controlled from the input chamber. A possible layout of the valve‐actuator integration is given in Figure S19 (Supporting Information). Both the rigid input and output chambers can be replaced with thick soft walls by molding.^[^ ^29^ ^]^ The integration would only require the merging of both molds, that of the bi‐shell valve and that of the soft actuator.

While our current concept is devised mainly to attain rapid deflation, the potential to embed more functions is at hand. For example, the bi‐shell valve can be designed as a fuse of volume change by placing the output chamber above the imperfect shell, rather than on the spherical cap (Figure S17, Supporting Information). In this case, the volume output will initially increase with the volume input until a threshold is reached; at this stage the imperfect shell inflates with the snapping of the valve. Another example is the potential to swap deflation (negative pressure) with inflation (positive pressure). Several pneumatic robots are actuated by inflation rather than deflation. By flipping the imperfect shell and the spherical cap upside down (Figure S17, Supporting Information), our bi‐shell valve can be reset to operate under inflation. Compared to existing valves^[^ ^43^, ^45^, ^53^ ^]^ that are capable of continuous operation, the bi‐shell valve and the variational designs for inflation here presented can provide an impulse output with finite volume (Figure 4C). At the design stage, it is necessary to ensure that such an impulse can provide the volume change that is sufficient to fast actuate the soft actuator. Moreover, while the bi‐shell valve presented here provides a simple task for fast actuation, more complex logic functions could be achieved by integrating the valve into the fluidic system of circuits of a soft robot.^[^ ^22^, ^23^, ^29^, ^44^ ^]^

## 3 Conclusion

To achieve rapid actuation of soft pneumatic actuators, we have introduced a soft bi‐shell valve that can convert a slow volume input into a fast output. The underpinning principle is the interaction during snap‐through of its elastic soft constituents: an imperfect shell and a spherical cap. Upon deflation, the former first stores sizeable values of volume change and elastic energy, which are then suddenly released upon snapping of the latter. Its rapid volume output can be used to deliver fast actuation. Upper bounds and performance metrics have been presented to design the bi‐shell valve for target requirements of soft robotic applications. The spherical cap determines the snapping pressure and the upper bounds of volume change and released energy, while the imperfect shell interacts with the cap to yield an attainable valve output. Tuning defect geometry and shell shape enables to passively code the snapping event, calibrate volume and energy output and maximize the valve performance. Through the demonstration of the striker, we have shown that the bi‐shell valve can accelerate the motion of soft actuators under volume control, thus avoiding the need for fast volume input provided by pressure control nor additional modifications to the body of the actuator that use chemical explosion or elastic instability. In conclusion, the bi‐shell valve concept introduced in this work along with its variational designs are poised to offer new routes to provide actuation of soft pneumatic actuators.

## 4 Experimental Section

### Methods

Section S1 in the Supporting Information details the fabrication methods, while the experimental characterization of the bi‐shell valve and the elastic shells are described in Section S2 in the Supporting Information. The effect of air compressibility is discussed in Section S3 (Supporting Information). Section S4 (Supporting Information) reports the numeric analysis. Sections S5 to S7 (Supporting Information) discuss the buckling modes of the imperfect shell, the interaction between the spherical cap and the imperfect shell, and the upper bounds of valve output. The flowchart detailing the valve design as well as design variations of the bi‐shell concept are presented in Sections S8 and S9 (Supporting Information). The effects of material property are studied in Section S10 (Supporting Information). Section S11 (Supporting Information) presents a possible layout for the integration of the bi‐shell valve into a soft robot or actuators, and Section S12 (Supporting Information) discusses the scenario of a similar valve with only one shell.

## Conflict of Interest

The authors declare no conflict of interest.

## Supporting information

Supporting Information

Supplemental Movie 1

Supplemental Movie 2

Supplemental Movie 3

Supplemental Movie 4

Supplemental Movie 5

## Data Availability

Data available on request from the authors.
