# Supplementary material for: Bi‐Shell Valve for Fast Actuation of Soft Pneumatic Actuators via Shell Snapping Interaction
Source: Adv Sci (Weinh). 2021 Jun 1;8(15):2100445. doi: 10.1002/advs.202100445 (PMC8336518; doi:10.1002/advs.202100445)
Supplement: Supplementary file 1 — Supporting Information [file ADVS-8-2100445-s005.pdf]

© 2021 Wiley-VCH GmbH

ADVANCED SCIENCE
Open Access

Supporting Information

for *Adv. Sci.,* DOI: 10.1002/advs.202100445

Bi-shell valve for fast actuation of soft pneumatic actuators via shell snapping interaction

*Chuan Qiao, Lu Liu, Damiano Pasini**

Supporting Information

Bi-shell valve for fast actuation of soft pneumatic actuators via shell snapping interaction

*Chuan Qiao, Lu Liu, Damiano Pasini**

S1. Fabrication

S1.1. Fabrication of the input chamber

Figure S1 illustrates the manufacturing process for the input chamber encompassing six layers laser cut (CM1290 laser cutter, SignCut Inc., Canada) from a 6-mm-thick acrylic plate (McMaster-Carr, USA). The first layer at the bottom is fully solid with geometry parallel to the external profile of the two shells. From the second to the fifth layer the acrylic plates consists of a 15.5-mm-wide ring with an external profile identical to the first layer, forming the wall of the input chamber. Moreover, the third and fourth layers have an opening at both ends along their long axis, a feature that allows connection to the PVC plastic tubing for volume input and pressure sensor. The sixth layer at the top covers the input chamber enclosed by the first five layers, with two large circular holes that connect the imperfect shell and the spherical cap. In addition, six smaller holes on the top layer are used to fasten the input chamber with other parts of the bi-shell valve. Adhaero SuperGlue (Dollarama, Canada) is used to join all pieces of the input chamber assembly, and silicone rubber Elite double 32 (Zhermack, Italy) is applied to seal off its internal walls.

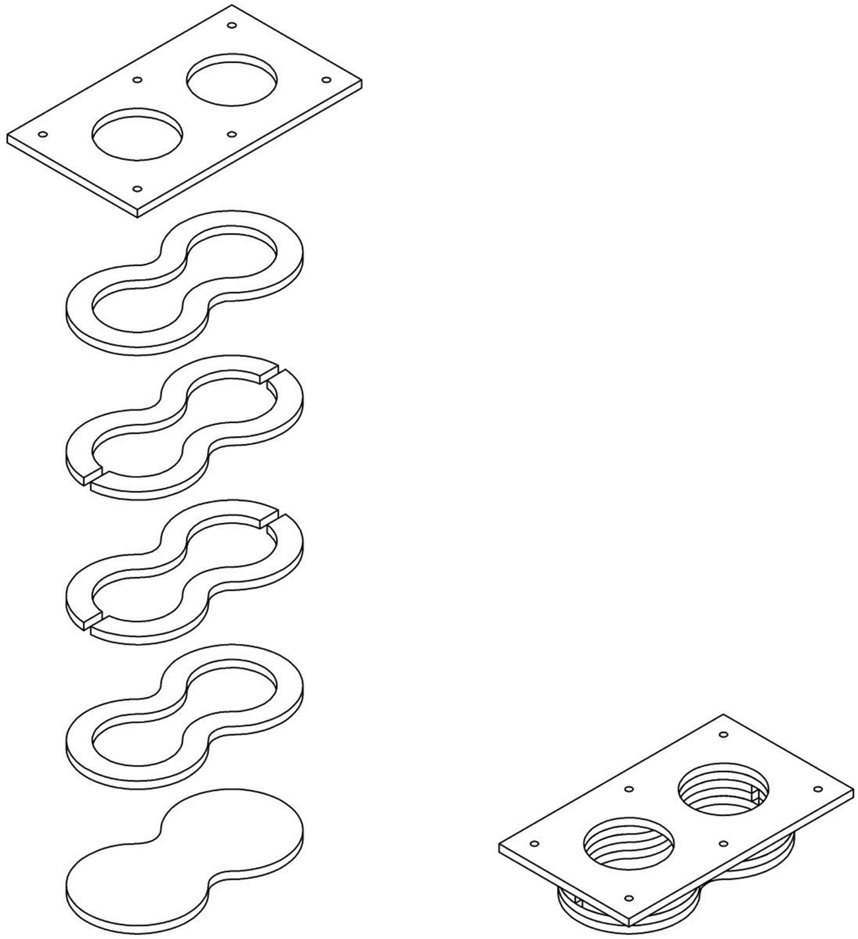

Figure S1. Assembly of the input chamber

S1.2. Fabrication of the output chamber

Figure S2 shows the output chamber consisting of five laser cut acrylic plates, which forms an open cubic cell that can capture the rapid volume change of the spherical cap. Again Adhaero SuperGlue (Dollarama, Canada) is applied to join the fives plates and Adhaero epoxy (Dollarama, Canada) is used to seal them. The small hole on the left-hand side of the cell provides the outlet of the output chamber, which in turn is connected through tubing to the soft actuator.

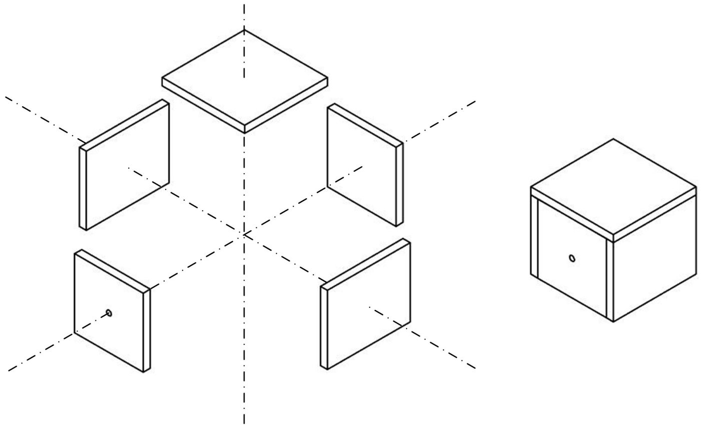

Figure S2. Assembly of the output chamber

S1.3. Fabrication of the elastic shells

Shell fabrication for both the imperfect shell and the spherical cap follows a procedure previously used.[1, 2] Elite Double 32 (Zhermack, Italy) is casted on the surface of 3D-printed molds (Figure S3). For each shell, we fabricated a 1 mm-thick mold with Onyx filament using fused deposition modeling (FDM). The surface of the molds has geometry identical to that of the as-designed elastic shells ($R = 25$ mm, $h/R = 0.2$, $\theta_L = 20°$, and $\theta_U = 85.9°$), with a groove at the bottom to collect excessive deposition of liquid and form a thick band that provide a clamping action. To locally reduce excessive accumulation of Elite Double 32 liquid on the curved surface of the mold, we used a homemade spin coating unit to spin the mold at a speed of ~240 rpm. More specifically, the shells were fabricated at room temperature by following these steps:

a) Prepare the catalyst and base of Elite Double 32 with 1:1 volume fraction.

b) Mix and manually stir the prepared catalyst and base for ~1 min.

c) Turn on the power of the spin coating unit.

d) Slowly pour the mixed Elite Double 32 solution onto the mold.

e) Spin ~10 min to remove excessive liquid on the mold.

f) Turn off the power of the spin coating unit.

g) Wait about 15 min to fully cure the Elite Double 32 solution.

h) Systematically increase shell thickness by repeating the above steps a) to g) until the designed thickness is attained.

i) Repeat steps a) and b) and fill the groove at the bottom of the mold with mixed Elite Double 32 solution.

j) Wait ~25 min until the Elite Double 32 solution is fully stabilized.

k) Peel off the elastic shell from the mold.

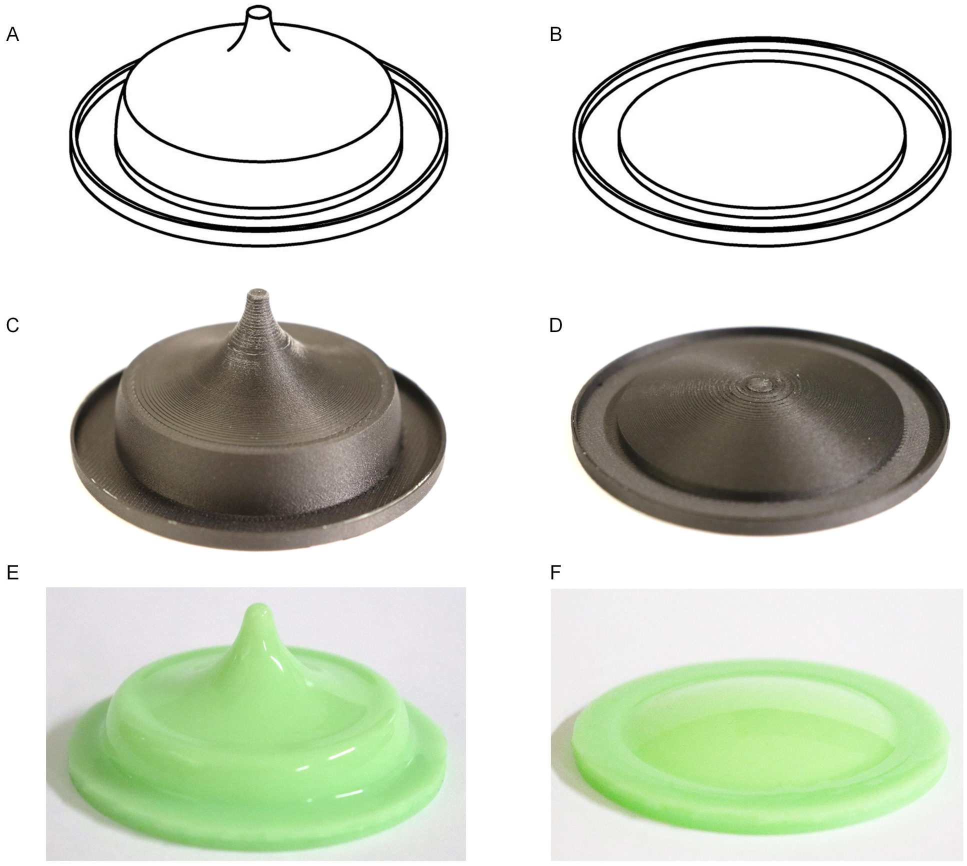

Figure S3. Mold for the imperfect shell and the spherical cap. (A) and (B): schematic of each respective mold. (C) and (D): 3D printed mold. (E) and (F): Fabricated shells.

S1.4. Assembly of the bi-shell valve

Figure S4 shows the components and assembly of our bi-shell valve system. The imperfect shell and the spherical cap are mounted on the input chamber, with their inner volume connected through the two large holes on the top surface of the input chamber. Any leakage that may exist between the elastic shells and the input chamber are sealed with Elite Double 32. Above the elastic shells is a fixture plate, which can be fastened to the input chamber with screws through six small holes along its edges. This fixture plate has two large circular holes that allow the elastic shells to freely deform without entering in contact with the fixture plate. Since the base of the spherical cap is thicker than that of the imperfect shell, the fixture plate can tightly clamp the base of the spherical cap on the input chamber to ensure airtight connection to the output chamber. The output chamber is mounted on the fixture plate with a square gasket made of Elite Double 32 that can tolerate the deformation of the fixture plate due to clamping. The leaks that may occur between the fixture plate and the output chamber are sealed with Elite Double 32 and Adhaero 5 minute epoxy (Dollarama, Canada).

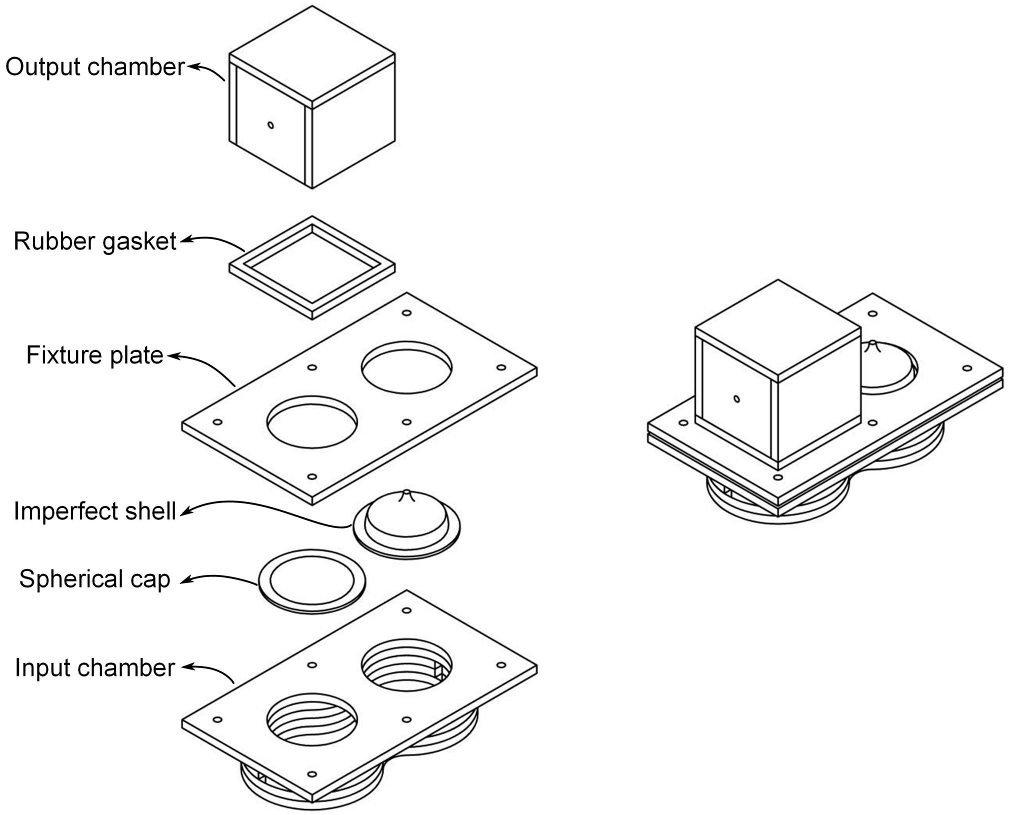

Figure S4. Components and assembly of the bi-shell valve system.

S1.5. Fabrication of the pneumatic striker

Our pneumatic striker consists of two main components: a miniature airbag to convert volume input into motion, and a paper stick to hit the table tennis ball. The airbag is cut from a 0.02-mm-thick compostable kitchen bag (no name, Loblaws Inc, Canada) as a folded bi-layer film with an area of 28x28 mm. A segment of tubing is glued on the bottom surface of the cut film with Adhaero SuperGlue. A hole is then cut through the film to enable airflow between the airbag and the tubing. Finally, the airbag is sealed with Elite Double 32. The paper stick is cut from a 0.29-mm-thick paper and then glued on the top surface of the airbag with Elite Double 32. The length of the stick is 56 mm, and the width is 7 mm.

S2. Experimental characterization

S2.1. Characterization of a single shell

To characterize the pressure-volume response of a single shell, we assemble the experiment apparatus shown in Figure S5, which includes an acrylic fixture to hold the sample, a polypropylene syringe of 60 mL capacity to extract air from the sample, a Bose ElectroForce 3510 tester (Bose Corporation, Framingham, Massachusetts) to control the syringe, a pressure sensor (HSCDRRN002NDAA5, Honeywell, USA) that has a measurement range of ±498 Pa and an accuracy of ±2.5 Pa, and a microcontroller (Arduino UNO, Arduino, Italy).

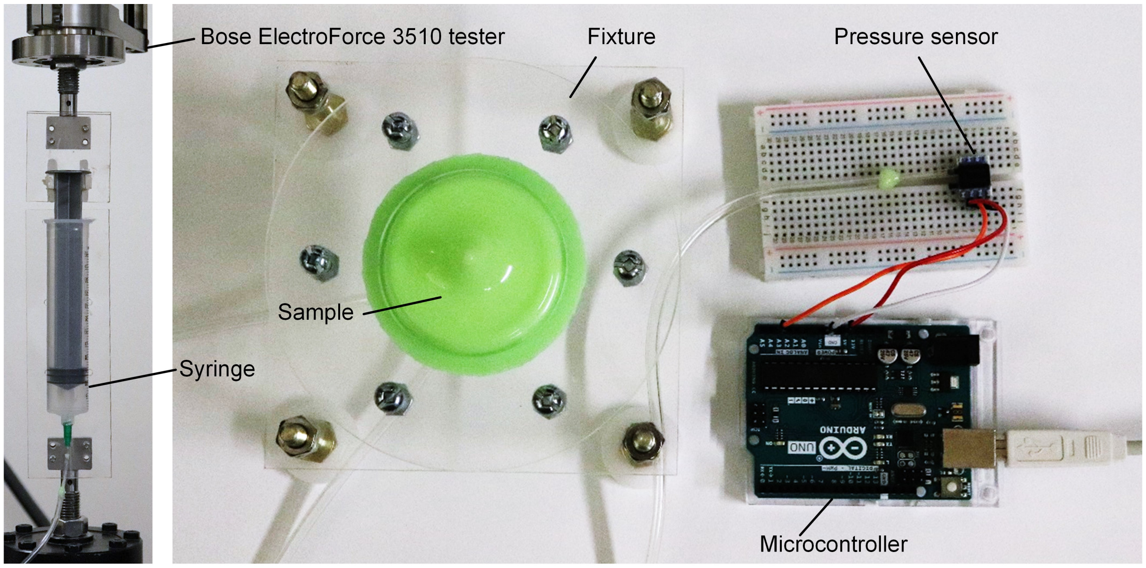

Figure S5. Experimental set-up for the characterization of a single shell.

The fixture consists of a top plate and a bottom plate. The top plate has a round geometry and is placed above the shell. There is a circular hole at the center, which not only allows the body of the shell to freely deform without entering in contact with the plate, but also clamps the base of the shell on the bottom plate. The bottom plate features a square geometry, and is connected to the syringe and the pressure sensor with a PVC plastic tubing. A 6-mm-thick ring gasket made of acrylic is glued at the center of the bottom plate, providing room for the spherical cap to fully deflate without getting into contact with the bottom plate. The top and

bottom plates can be tightly fastened with six equally spaced screws to prevent any air leakage.

In our test, the syringe is pulled by the Bose tester at a speed of 0.1 mm s^{-1} to generate a constant flow rate of 3 mL min^{-1}. The microcontroller is programmed to read the pressure measurement at a frequency of 20 Hz, which is then recorded as a function of time with a data acquisition software (PLX-DAQ, Parallax, USA). For both the spherical shell and the imperfect shell, three samples have been tested under identical conditions. The resulting curves (pressure-volume) along with the representative envelope of the experimental response of all tested samples are plotted in Figure 2E and 2F.

S2.2. Characterization of the bi-shell valve

Our experiment with the bi-shell valve is performed with the set-up described above for deflation and pressure measurement. The syringe and the pressure sensor are connected to the input chamber with the tubing at the bottom the valve. The flow rate of deflation is 3 mL min^{-1}, while the frequency of pressure measurement is 20 Hz. To assemble the bi-shell valve, we select one pair of spherical cap and imperfect shell from the pool of the single shell samples tested individually in the analyses of the previous sections. The result from the tests are shown in Figure S6. The results show that the snapping pressure of the valve can be programmed by the clamping condition of the spherical cap from 405 Pa when the spherical cap is not clamped (test 1), to 591 Pa when the spherical cap is tightly clamped (test 4). The increase in snapping pressure is caused by the initial deformation of the spherical cap due to clamping, a behaviour studied numerically in Supporting Information S4.1. The curve of test 2 corresponds to the results illustrated in Figure 2D with a snapping pressure identical to the test result of the individual spherical cap shown in Figure 2E. We assume that the initial deformation of the spherical cap in test 2 is identical to the initial deformation of the cap

when tested alone. The curve of test 3 corresponds to the bi-shell valve used in our demonstrative experiment, where the spherical cap buckles at 549 Pa. The higher snapping pressure of 591 Pa attained in test 4 is only used to show the role of a given clamping condition.

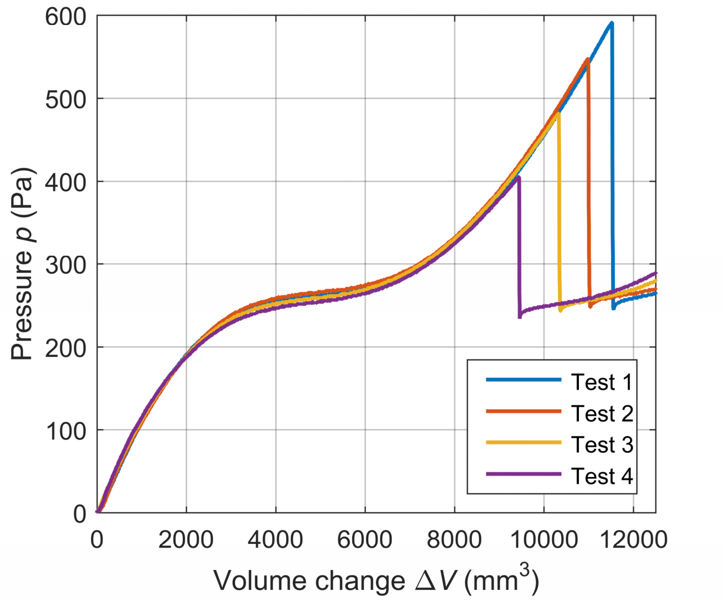

Figure S6. Pressure-volume response of the bi-shell valve. Pressure-volume response of the bi-shell valve. The spherical cap is not clamped in test 1 and tightly clamped in test 4, while tests 2 and 3 correspond to intermediate clamping conditions.

S3. Effect of air compressibility

Since air is a compressible fluid, here we study the influence of air compressibility. We assume that the air in the bi-shell-valve, the tubing, and the syringe follows the ideal gas law:

$$pV = nR_gT \tag{S1}$$

where p is the pressure of the gas, V is the volume of the gas, n is the amount of substance of gas, R_g is the ideal gas constant, and T is the absolute temperature of the gas. In both the

initial state and loaded states. the gas in the pneumatic system (the bi-shell valve, syringe and connecting tubing) must satisfy

$$p_{sys0}V_{sys0} = nR_g T_{sys0} \tag{S2}$$

and

$$p_{sys1}V_{sys1} = nR_g T_{sys1} \tag{S3}$$

where the subscript stands for initial (0) and loaded states (1).

We assume the gas undergoes an isothermal process ($T_{sys0} = T_{sys1}$), which yields

$$p_{sys0}V_{sys0} = p_{sys1}V_{sys1} \tag{S4}$$

The volume change due to air compressibility can be expressed as

$$\left|V_{sys1} - V_{sys0}\right| = \left|\frac{p_{sys0}}{p_{sys1}} - 1\right| V_{sys0} \tag{S5}$$

This value is proportional to the initial volume of the system V_{sys0}, and increases with the pressure change from the initial state. In our bi-shell valve, the maximum change in pressure from the initial state (atmosphere pressure $p_{sys0} = p_{\text{atm}} = 1.01 \times 10^5$ Pa) is below 550 Pa, which yields

$$\left|\frac{p_{sys0}}{p_{sys1}} - 1\right| < 0.54\% \tag{S6}$$

We can thus rewrite the volume change due to air compressibility as

$$\frac{\left|V_{sys1} - V_{sys0}\right|}{V_{sys0}} < 0.54\% \tag{S7}$$

For our tests on the bi-shell valve, the total volume is about $V_{sys0} = 1.8 \times 10^5 \text{ mm}^3$, leading to a volume change of $\left|V_{sys1} - V_{sys0}\right| < 9.9 \times 10^2 \text{ mm}^3$. For the characterization of a separate shell, the total volume of the system is about $V_{sys0} = 5 \times 10^4 \text{ mm}^3$, leading to a volume change of

$\left|V_{sys1} - V_{sys0}\right| < 2.7\times10^{2}\ \mathrm{mm}^3$. These values are tiny in comparison with the volume change of the shells ($\sim 1.3\times10^{4}\ \mathrm{mm}^3$ for the bi-shell valve and $\sim 7.5\times10^{3}\ \mathrm{mm}^3$ for a single shell), and corroborate the assumption made in this work that air compressibility can be neglected. Hence, the volume change of the syringe can be assumed as the volume change of the bi-shell valve and the separate shells.

S4. Finite element analysis

To further investigate the mechanical performance of the bi-shell valve, we conduct a set of finite element method (FEM) simulations with the commercial software package ABAQUS/STANDARD. The shell material is modelled as an incompressible neo-Hookean solid. The Young's modulus and Poisson's ratio (1.23 MPa and 0.5) are determined by fitting the simulation results with the experimental data within a range previously used in the literature.[1, 3] This leads to the adoption of the following coefficients for our neo-Hookean model: $\mathrm{C10} = 0.205\ \mathrm{MPa}$ and $\mathrm{D1} = 0\ \mathrm{MPa}^{-1}$. We employ the modified Riks method to simultaneously solve for pressure and shell deformation. Since in our experiments we observe that both the imperfect shell and the spherical cap exhibit only an axisymmetric mode of deformation, we build our numerical model with axisymmetric elements (the two-node linear shell element SAX1 or the four-node bilinear quadrilateral element CAX4RH) to avoid the expensive computational cost of three-dimensional simulations. Although in some cases the imperfect shell may exhibit non-axisymmetric deformations, our previous study shows that an axisymmetric analysis can still be sufficient to retain a high level of accuracy.[2] We impose a fixed boundary condition at the bottom of the shells and a uniform pressure at their surfaces. The volume change ΔV is calculated with the pressure p and the total external work done by the pressure U_p, which is given by

$$\Delta V = \int \frac{1}{p} \mathrm{d}U_p. \tag{S8}$$

As described below, our computational analysis for each separate shell as well as for the bi-shell valve is conducted into two steps. First, we consider an as-designed (ideal) model that is free from any manufacturing imperfections and does not account for any initial deformation caused by the clamping of the bottom ring. In this scenario, we systematically explore the geometric space of the bi-shell valve to unveil its sensitivity to a varying shell geometry. Second, to validate our numerical model with experimental results, we develop a set of realistic models, one for the spherical cap and the other for the imperfect shell. These models enable to capture the effect of the initial deformation due to clamping in spherical cap, and to incorporate as-manufactured imperfections, in particular thickness variations, in fabricated imperfect shells.

S4.1. Modelling of the spherical cap

As-designed model. The as-designed spherical cap is modelled with axisymmetric line element SAX1. The geometry of the spherical cap is $t_1/R = 0.05$, $h/R = 0.2$, and $R = 25$ mm. A mesh convergence study shows that 51 elements are sufficient to model the spherical cap (Figure S7A). In this work, around 51 elements are used for the spherical cap. To systematically study the response of the spherical cap with varying geometry, we explore the geometry space defined by the normalized thickness t_1/R ranging from 0.01 to 0.1 and the normalized height h/R spanning from 0.1 to 0.5; the radius at the base is fixed as $R = 25$ mm.

Realistic model. To capture cross-section variation in a representative sample of the spherical cap, we use a digital camera EOS 800D (Canon, Japan). Our observations show that the

spherical cap has a uniform thickness profile (Figure S8A), hence our analysis of the spherical cap studies only the role of the initial deformation due to clamping.

In Figure S9A, the spherical cap is modelled with CAX4RH elements, whereas the acrylic plate that clamps the base of the cap is modelled with rigid body line elements RAX1. For the spherical cap, our mesh convergence study shows that four elements through the thickness are sufficient (Figure S7B). Hence, we employ here at least four elements through the thickness.

The interaction between the cap and the plate is set as "hard" contact with a friction coefficient of 0.5. To investigate the effects of the initial deformation due to clamping, we first apply a downward displacement on the plate, and then apply a pressure on the shell to deflate the shell. The displacement is systematically varied from 0 (no clamping) to 0.3 mm (tight clamping). In Figure S9B, when the cap is clamped through the plate for 0.3 mm, an upward displacement occurs at the top of the cap. In Figure S10A, we find that the buckling pressure increases monotonically with the displacement of the clamping plate over a wide range of values from 403 Pa (no clamping) to 587 Pa (0.3 mm of clamping). From this set of results for the spherical cap, we decide to include the initial deformation due to clamping in our realistic numerical model. To minimize the difference of results between experiments and simulations, the displacement due to clamping is set as 0.1026 mm. This enables to yield a buckling pressure close to that of the representative sample of the spherical cap (Figure 2E), and to bring below 0.6% the relative error in the buckling pressure between simulation and experiment. In addition, we find that the buckling pressure of the unclamped case (Figure S10A) is slightly lower than the results in Figure S7. The reason for this is that in the simulations that study the effect of clamping, the thick band at the base of the shell is included; this provides an elastic support to the shell which is dissimilar to the fixed boundary condition employed in other simulations.

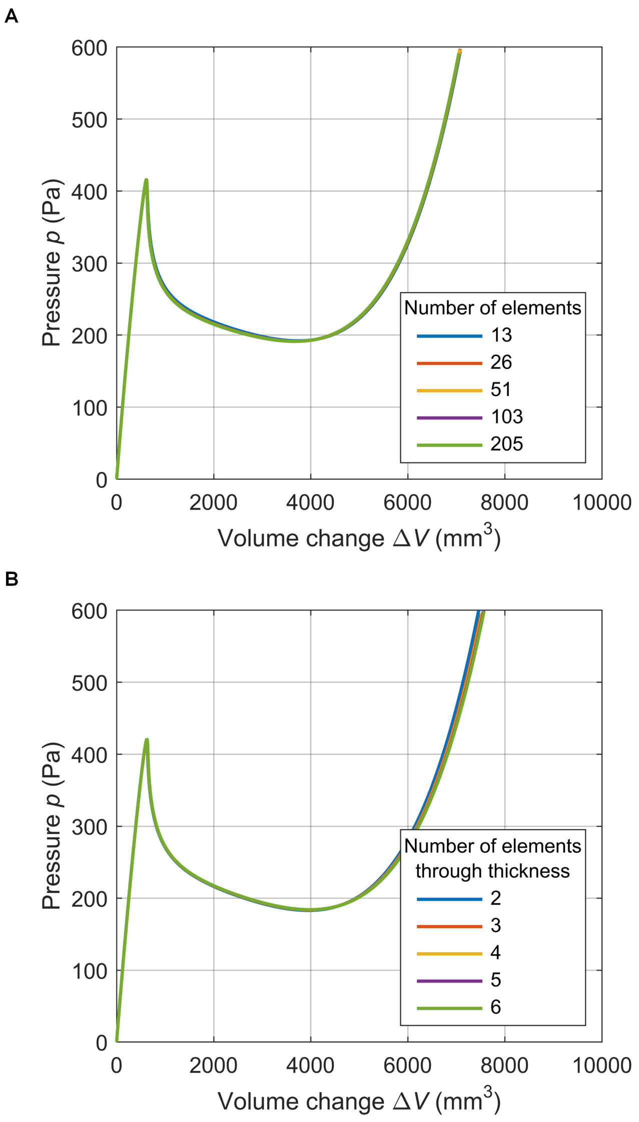

Figure S7. Mesh convergence study of the spherical cap. (A) SAX1 element. (B) CAX4RH element. Curves of different colors overlay, thereby showing that the simulation results for meshes with given number of elements have converged.

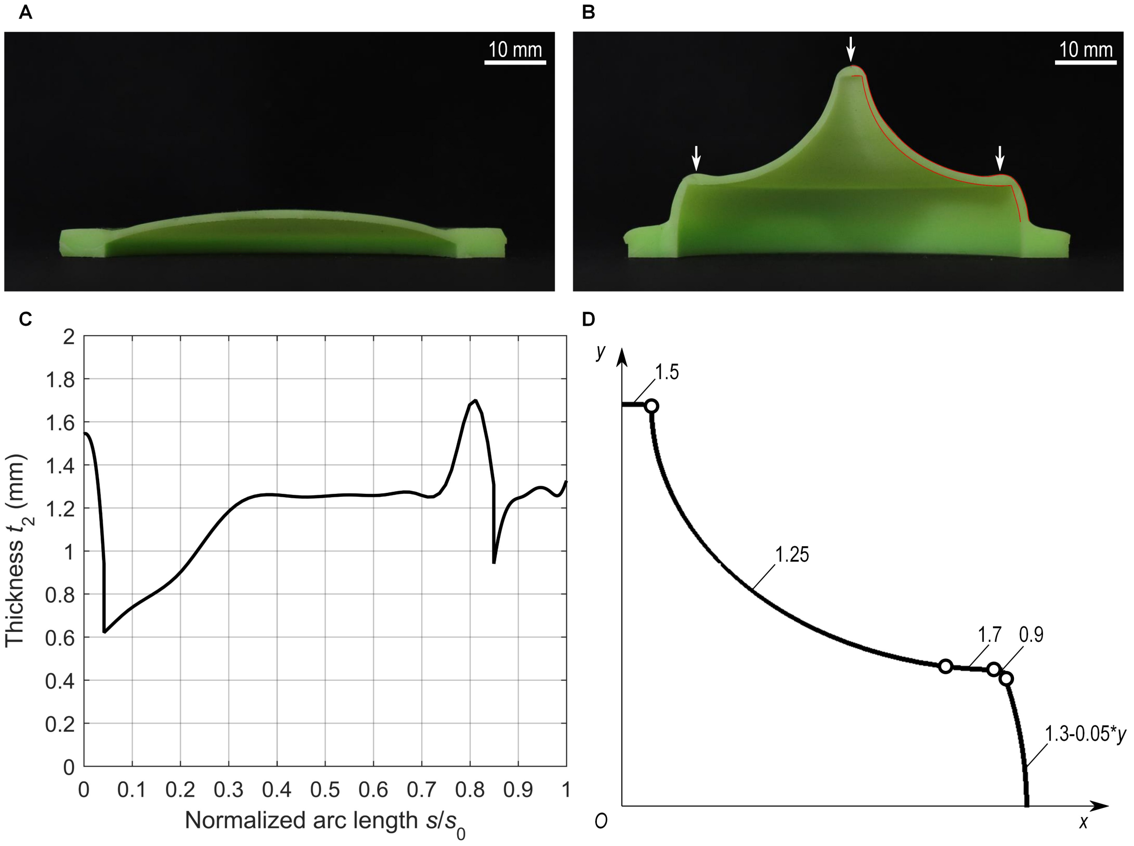

Figure S8. Cross-section of representative as-manufactured shell. (A) The spherical cap has a uniform thickness profile. (B) For the imperfect shell, on the other hand, the thickness profile is not uniform due to manufacturing. The red curves mark the upper and lower surfaces of half shell. The arrows marks the spots at the top of the shell and at the bottom of the imperfection with increased thickness. (C) Thickness t_2 of the imperfect shell measured as a function of the normalized arc length s/s_0 from the top ($s/s_0 = 0$) to the base ($s/s_0 = 1$). s and s_0 are the arc length and the total arc length. (D) Thickness profile of the imperfect shell used to generate the realistic computational model. The white dots partition the shell into five sections; to each of them the shell thickness is assigned separately to approximate the thickness distribution in (C). y is the distance from the base plane and the unit of the thickness is millimeter.

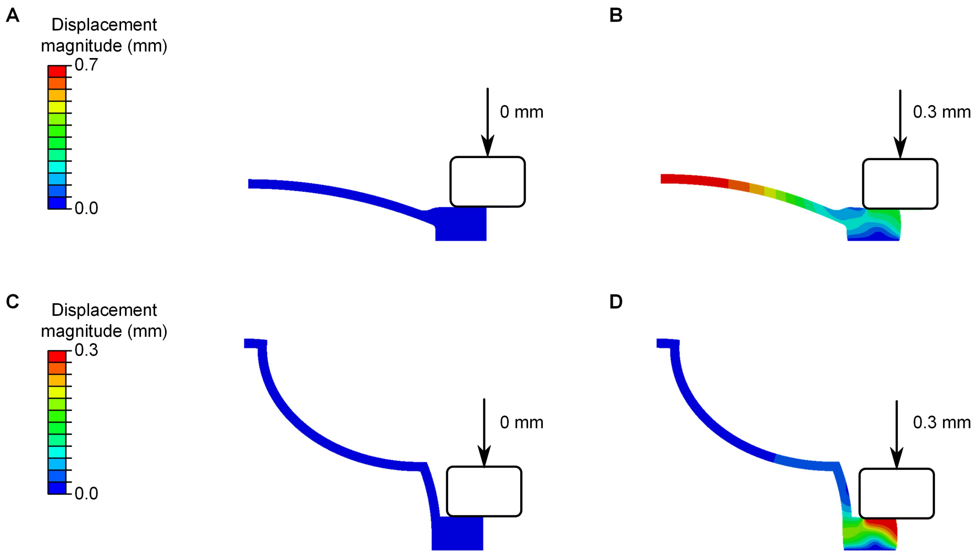

Figure S9. Deformation of the shells due to clamping. (A) Initial and (B) clamped configuration of the spherical cap. (C) Initial and (D) clamped configuration of the imperfect shell.

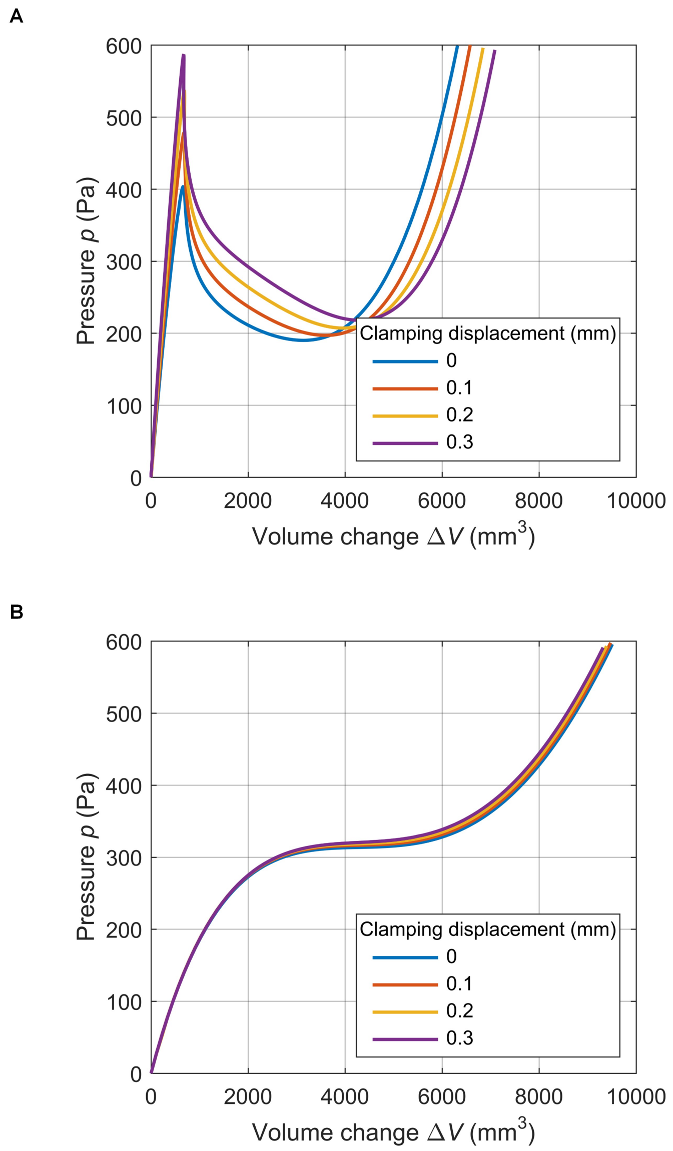

Figure S10. Pressure-volume responses of clamped shells. (A) spherical cap, and (B) imperfect shell.

S4.2. Modelling of the imperfect shell

As-designed model

We model the as-designed imperfect shell using the axisymmetric shell element SAX1. The geometry of the imperfect shell is defined by the following parameters: $t_2/R = 0.05$, $\theta_{\mathrm{L}} = 20°$, $\theta_{\mathrm{U}} = 85.9°$, and $R = 25$ mm . A wide design space is explored to investigate the

buckling sensitivity to the as-designed defect in the form of an ellipse, with respect to the normalized thickness ($0.02 \le t_2/R \le 0.1$) and meridional angle at the upper and lower boundary of the defect ($20° \le \theta_\mathrm{L} \le \theta_\mathrm{U} \le 85°$). A mesh convergence study shows that 81 SAX1 elements are sufficient to model the imperfect shell (Figure S11A). In this work, an average of 81 elements are used for the imperfect shell.

Realistic model

As described above, our fabrication process, in particular the mould we used to produce our samples, had the following outcome on the shell geometries. The thickness profile of the spherical cap is uniform since the mould has no change in curvature, as opposed to that of the imperfect shell, which has variations due to abrupt changes in the curvature of the mould. To develop a realistic model of the imperfect shell with response that parallels that of the as-manufactured geometry, we investigate separately the role of non-uniform thickness as well as that of the initial deformation due to clamping, as described below.

Non-uniform thickness profile. Figure S8B shows that the as-manufactured shell features a thickness build up at locations above and below the elliptical arc; it is at those points that sudden changes of curvature appear in the mould. To obtain precise measurement of the thickness profile of the imperfect shell, we determine the distance between the inner and outer surfaces of the shell first from digital images (e.g. Figure S8B), and then by rectifying the data with measurements taken through a digital caliper (Figure S8C). This set of results is used to generate a numerical model that captures thickness variations along the shell profile; in particular the thickness profile of the shell is partitioned into five sections (Figure S8D), and to each of these portions the pertinent thickness is assigned. The SAX1 element is used to generate the model.

Initial deformation due to clamping. Here we solely study the role of the initial deformation due to clamping on an imperfect shell with uniform thickness. Figure S9C shows an imperfect

shell clamped to the fixture plate. The plate is modelled as a rigid body with the axisymmetric rigid two-node line element RAX1. Since the clamped base of the imperfect shell is too thick to be considered as a shell, we use the axisymmetric quadrilateral element CAX4RH instead of the SAX1 element. Our mesh convergence study shows that four elements through the thickness are sufficient for the imperfect shell (Figure S11). Hence, at least four elements through the thickness are adopted. The interaction between the shell and the clamp is set as "hard" contact with a friction coefficient of 0.5. In our simulations, we first simulate the initial deformation by imposing a vertical displacement on the plate, and then apply a pressure on the shell to deflate the shell. The displacement of the plate is varied from 0 to 0.3 mm. Figure S9D shows that the deformation due to clamping is localized at the base, while the body of the shell is not affected. In Figure S10B, the response of the imperfect shell is also not sensitive to the initial deformation caused by clamping. From these results, we conclude that it is reasonable to neglect the initial deformation of the imperfect shell due to clamping.

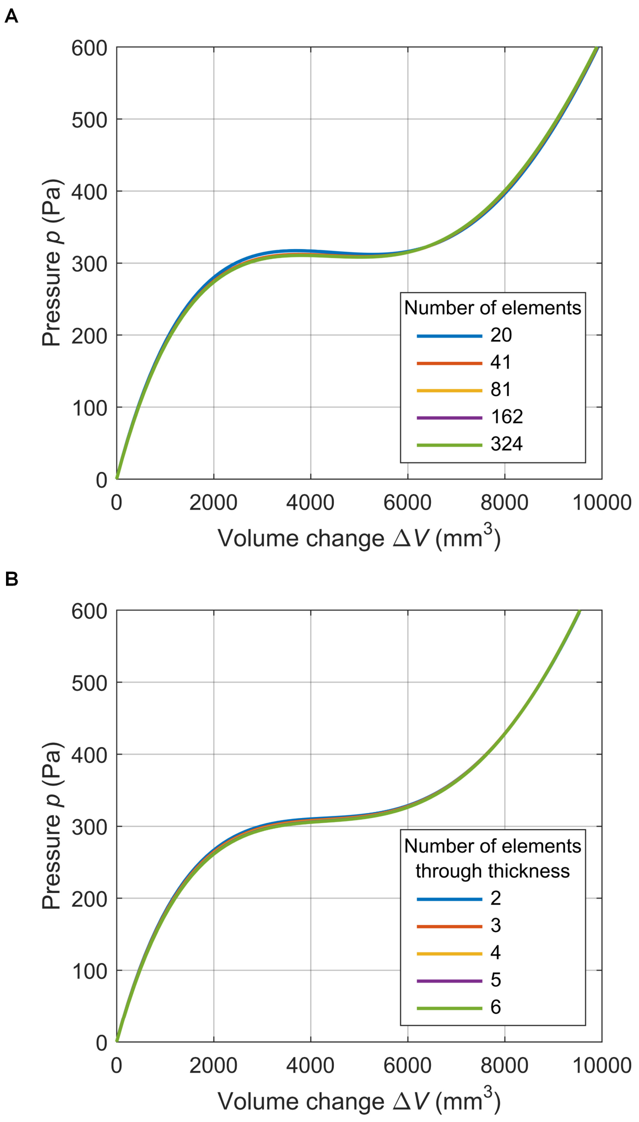

Figure S11. Mesh convergence study of the imperfect shell. (A) SAX1 element. (B) CAX4RH element. Curves of different colors overlay, thereby showing that the simulation results for meshes with given number of elements have converged.

S4.3. Modelling of the bi-shell valve

We now study the collective response of the shells forming our valve system by combining the models of each individual shell into one model. SAX1 elements are used for both constituent shells of the as-designed model, which does not feature any variation in thickness

or initial deformation due to clamping. On the other hand, for the realistic model, the thickness variation in the imperfect shell and the initial deformation of the spherical cap are modelled as described above for the realistic model of each shell.

We impose a uniform pressure on both shells, and trace the equilibrium path of the bi-shell system with the modified Riks method. The released energy is calculated as the negative of the area under the pressure-volume curve

$$\Delta U^{*} = -\int_{S} p \mathrm{d}V \tag{S9}$$

where S is the equilibrium path from the pre-snapping state (i) to the post-snapping state (ii) (Figure 1).

S5. Buckling modes of imperfect shell

While the buckling of the spherical cap subject to uniform pressure has been extensively studied in literature,[1, 4, 5] only recently we unveil that of an imperfect shell with a large axisymmetric defect away from the pole.[2] Therein, we investigated an individual imperfect shell with a circular defect, and demonstrated the existence of additional three buckling modes, besides to the classical bifurcation, that can be programmed on demand through geometry tuning.

In the current work, we amend the defect geometry to an elliptical arc for the convenience of manufacturing, and specify two defining parameters (Figure 4B): the meridional angles at the upper and lower boundary of the defect θ_{U} and θ_{L}. By varying θ_{U} and θ_{L}, we can show the emergence of four possible buckling modes (Figure S12). For a small defect (Figure S12A), the shell defect undergoes the classical bifurcation buckling, which is characterized by a downward dimple at the pole of the hemisphere. The pressure increases rapidly to a high buckling pressure (bifurcation point) before dropping immediately to a low plateau. We name this mode as mode 1.[2] When the defect size increases, Figure S12B shows that the buckling

mode changes from the classical bifurcation mode to a snap-through buckling mode, which is characterized by a localized deformation that evolves mainly within the defect (mode 2). Similar to mode 1, the pressure attains the maximum at a small volume change (limit point 1). For further increase of defect size (Figure S12C), the maximum pressure is reached when the main deformation localizes below the defect (mode 3). Dissimilar from mode 1 and mode 2, the pressure in mode 3 gradually increases to the maximum at a much larger volume change (limit point 2). Depending on the shell geometry, the pressure may also show a plateau before the attainment of the maximum pressure (Figure 1E). In a special case (Figure S12D), the shell buckles with a mixed mode that combines mode 2 and mode 3. The pressure shows a lower peak at a small volume change (limit point 1) before finally attaining the maximum value at a large change in volume (limit point 2).

The four buckling modes identified above can be overlaid onto the map of the attainable valve output illustrated in Figure 4. The result is shown in Figure S13. Here, the boundary (red line) is marked between mode 1 and 2, where the imperfect shell collapse immediately upon deflation, and the zone of mode 3 and 4, where the imperfect shell can undergo a large deformation before collapse. The region of mode 1 and 2 is on the lower right-hand side of the red line where θ_U and θ_L are close in value (small defect), whereas the zone of mode 3 and 4 is on the other side where the defect is large.

The map in Figure S13 helps gain essential insights into the role the shell defect plays on the performance of our bi-shell valve. In particular, the domain boundaries demonstrate that to maximize the valve output, values of θ_U and θ_L falling within the zone of mode 3 and 4 should be preferred, as opposed to those of the other zone (mode 1 and 2), where the valve output is practically null. The cause for the difference we observe here lies in the interaction between the spherical cap and the imperfect shell, as discussed in Section S6 of Supporting Information.

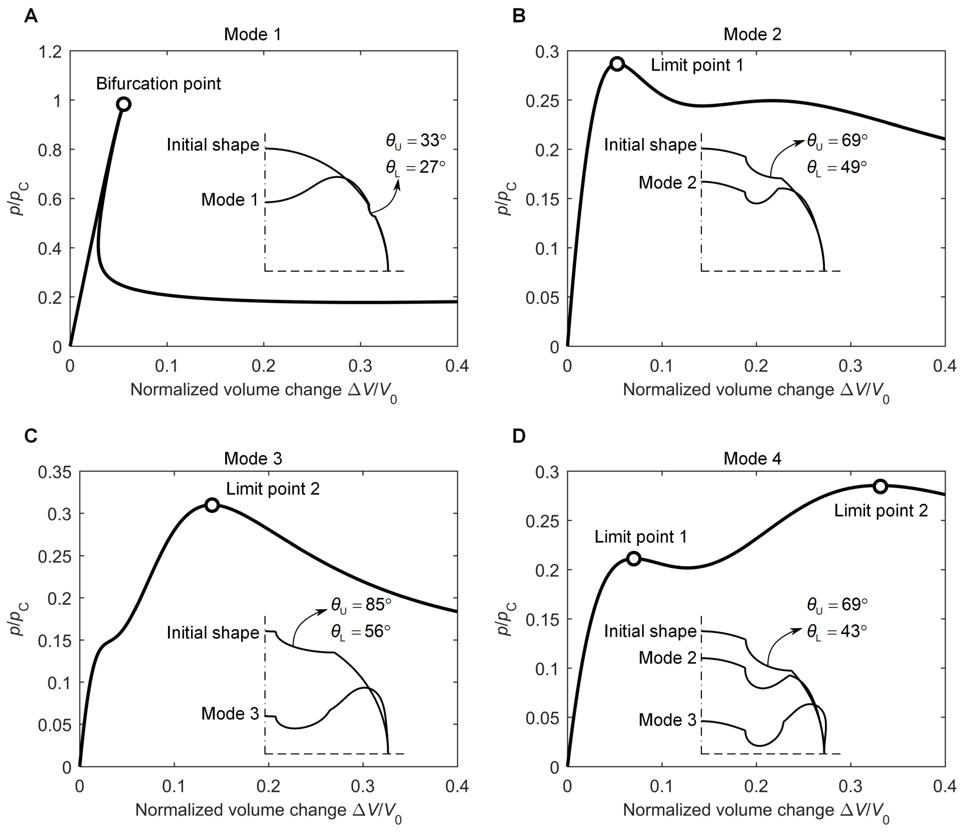

Figure S12. Possible deformation modes leading to shell collapse. (A) Mode 1: Bifurcation buckling with a dimple-like shape response. (B) Mode 2: Snap-through buckling 1 characterized by localized deformation within the imperfection. (C) Mode 3: Snap-through buckling 2 with localized deformation below the imperfection. (D) Mode 4: Snap-through buckling combining mode 2 and 3.

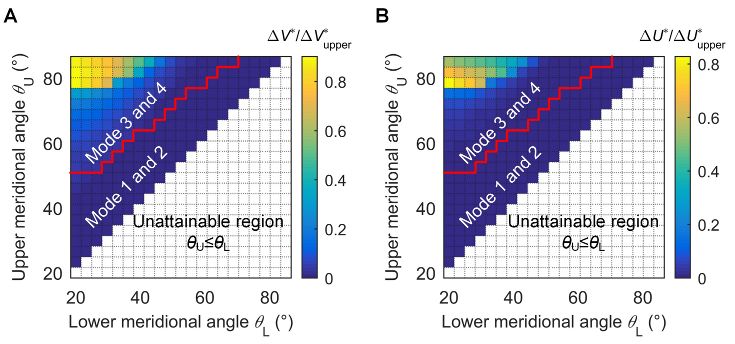

Figure S13. Attainable valve output with corresponding buckling modes. (A) Attainable volume change. (B) Attainable released energy. The red line marks the boundary between the zone of mode 1 and 2 and the zone of mode 3 and 4.

S6. Interaction between spherical cap and imperfect shell

S6.1. Interaction between shells

In our bi-shell valve, the total volume change of the imperfect shell and the spherical cap is determined by the input volume change dispense by the syringe. Since the air compressibility can be neglected (see S3), the sum of the volume change of each shell (ΔV_1 and ΔV_2) should balance that of the syringe (ΔV_{in}) such that

$$\Delta V_1 + \Delta V_2 = \Delta V_{\text{in}} \tag{S10}$$

When the imperfect shell and the spherical cap are slowly deflated by the syringe, their pressure at the quasi-equilibrium state must be equal

$$p_1\left(\Delta V_1\right) = p_2\left(\Delta V_2\right) \tag{S11}$$

where $p_1\left(\Delta V_1\right)$ and $p_2\left(\Delta V_2\right)$ are the pressure of each shell as a function of its own volume change. Substituting Equation S10 into Equation S11 yields

$$p_1\left(\Delta V_1\right) = p_2\left(\Delta V_{\text{in}} - \Delta V_1\right) \tag{S12}$$

At the pre-snapping state (i), Equation S11 and S12 are rewritten as

$$p_1\left(\Delta V_{1(\text{i})}\right) = p_2\left(\Delta V_{\text{in(i)}} - \Delta V_{1(\text{i})}\right) = p_2\left(\Delta V_{2(\text{i})}\right) \tag{S13}$$

where the subscript (i) denotes the pre-snapping state. While the pre-snapping state of the spherical cap is determined by its own buckling point, the pre-snapping state of the imperfect shell can be determined by finding the value of $\Delta V_{2(\text{i})}$ that satisfies Equation S13.

At the post-snapping state (ii), the balance of pressure is rewritten as

$$p_1\left(\Delta V_{1(\text{i})} + \Delta V^*\right) = p_2\left(\Delta V_{\text{in(i)}} - \left(\Delta V_{1(\text{i})} + \Delta V^*\right)\right) = p_2\left(\Delta V_{2(\text{i})} - \Delta V^*\right) \tag{S14}$$

where ΔV^* is the volume change due to snapping. Since $\Delta V_{1(\mathrm{i})}$ and $\Delta V_{2(\mathrm{i})}$ are already known from Equation S13, the post-snapping state (ii) can be determined by finding the volume change ΔV^* that satisfies Equation S14.

When the pre and post-snapping states have been determined, the released energy can be calculated from the separate response of each shell as

$$\Delta U^* = -\int_{\Delta V_{1(\mathrm{i})}}^{\Delta V_{1(\mathrm{ii})}} p_1 \mathrm{d}V - \int_{\Delta V_{2(\mathrm{i})}}^{\Delta V_{2(\mathrm{ii})}} p_2 \mathrm{d}V \tag{S15}$$

where $\Delta V_{1(\mathrm{ii})} = \Delta V_{1(\mathrm{i})} + \Delta V^*$ and $\Delta V_{2(\mathrm{ii})} = \Delta V_{2(\mathrm{i})} - \Delta V^*$ are the volume changes at the post-snapping states.

Since Equation S15 does not require handling the simulation of the whole valve, rather it allows to determine the global performance of the valve from those of the constituents. For this reason, the approach that we propose in this work enables a sizeable reduction of the computational cost, and can be readily used to compute the attainable valve output that is plotted in Figure 4.

S6.2. Relation between shell interaction and valve output

Equation S10 to S15 provide a mathematical description of the general interaction between the shells. However, they have been applied neither to assess the pressure volume curves of the individual shells nor to calculate the valve output. In this section, we apply these equations to analyze a group of pressure-volume curves with changing thickness so as to further explain the relation between shell interaction and valve output. Our specific focus is on understanding the reason for the small yellow regions in Figure 4, where the volume change and released energy reach the maximum values.

Figure S13 shows a set of the pressure-volume responses of the shells illustrated in Figure 4B. The curves pertain to a spherical cap (red line) and a an array of imperfect shells with varying thickness t_2/R (green and black lines). A range of responses can be observed.

- The green curves represent imperfect shells with $0.045 < t_2 / R < 0.055$. Here, the plateau pressure is located between the pre and post-snapping pressure p_{i} and p_{ii}, thereby allowing a large volume change at the plateau stage attained with a minor decrease in pressure resistance. This large volume change is a prerequisite for the shell to release a large amount of energy, hence to maximize the valve performance output. When the plateau pressure values gradually approaches p_{i} with increasing thickness t_2 / R , the released energy reaches a maximum value of $\Delta U / \Delta U_{\mathrm{upper}} = 0.70$ at $t_2 / R = 0.054$ with a sizeable volume change of $\Delta V / \Delta V_{\mathrm{upper}} = 0.81$.
- On the other hand, the black lines describe responses governed by $t_2 / R < 0.045$ and $t_2 / R > 0.055$. In this case, the plateau pressure is either lower or higher than both the pre and post-snapping pressure. Snapping occurs outside the plateau stage, thus causing a fast drop in pressure from p_{i} to p_{ii}. This set of imperfect shells (black) can only snap for a tiny volume change, and release a small amount of elastic energy as opposed to the imperfect shells in the range $0.045 < t_2 / R < 0.055$. Moreover, the thinnest shell with $t_2 / R = 0.02$ have a maximum pressure that is lower than the buckling pressure of the spherical cap. In this case, the bi-shell system cannot snap because the pressure is unable to reach the buckling pressure of the cap.

A comparison of Figure S12 with Figure S14 highlights that only mode 3 and 4 are suitable for the bi-shell valve. In mode 1 and 2, the pressure of the imperfect shell quickly reaches the maximum with a small volume change. On one hand, if the maximum pressure is larger than the buckling pressure of the spherical cap, the shell interaction will be similar to the case in Figure S14 for thick imperfect shells with $t_2 / R > 0.055$ (black lines on the left). The bi-shell valve can only snap for a small volume change and released energy. On the other hand, if the maximum pressure is less than the buckling pressure of the spherical cap, there will be no

snapping at all, as in the case with the thinnest imperfect shell ($t_2 / R = 0.02$). In mode 3 and 4, the imperfect shell can undergo a large deformation before attaining the maximum pressure, a response that can potentially form a plateau of pressure before collapse. The green lines in Figure S14 therefore identify the set of bi-shell valves that can attain a large volume change when the plateau pressure is between the pre and post-snapping states; in addition, their maximum values of released energy can be obtained when the plateau pressure is just below the pre-snapping pressure.

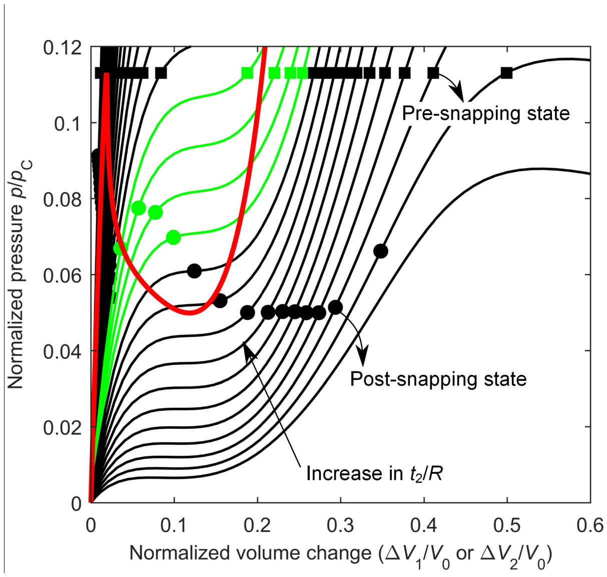

Figure S14. Pressure-volume response of the shells in Figure 4B. The red curve represents the spherical cap. The green lines describe imperfect shells in the range of $0.045 < t_2/R < 0.055$, while the black curves pertain to the remaining set of imperfect shells ($0.02 \le t_2 / R < 0.045$ and $0.055 < t_2 / R \le 0.1$). The square and circular dots mark the pre and post-snapping states of each imperfect shell.

S7. Upper bounds of valve output

Here we study the upper bounds of our valve output for both volume and released energy.

Figure S15 shows two representative curves of pressure-volume, each representing one individual shell, the spherical cap (A) and the imperfect shell (B). Upon snapping, the

pressure of both shells decreases from p_{i} to p_{ii}. In ideal conditions, we could assume the post-snapping pressure of the imperfect shell retains the pre-snapping pressure p_{i} (dashed line in Figure S15B). Thus, the pressure of the spherical cap could also retain p_{i}, thus leading to a post-snapping volume change of $\Delta V_{1\mathrm{(iii)}}$ (Figure S15A). Since it is unrealistic for the imperfect shell to have a post-snapping pressure higher than p_{i}, the post-snapping volume change of the spherical cap will never get larger than $\Delta V_{1\mathrm{(iii)}}$. Thus, $\Delta V^*_{\mathrm{upper}} = \Delta V_{1\mathrm{(iii)}} - \Delta V_{1\mathrm{(i)}}$ is the upper bound for the volume change output.

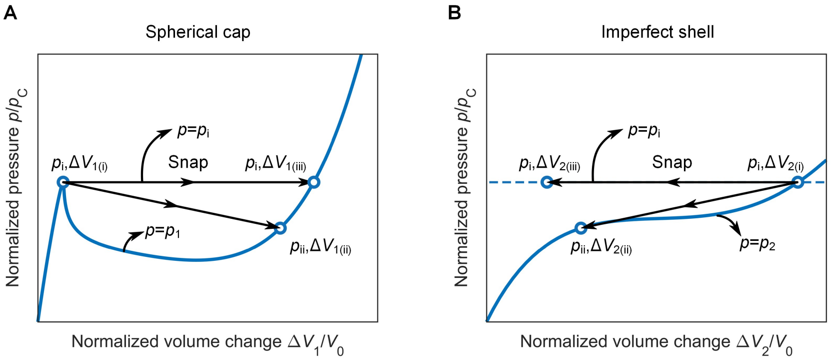

Figure S15. Representative pressure-volume responses of individual shells. (A) Spherical cap. (B) Imperfect shell. The dashed line in (B) shows an ideal scenario describing the case where the pressure of the imperfect shell does not drop after snapping; this phenomenon leads to state (iii) with pressure p_{i} and volume change $\Delta V_{2\mathrm{(iii)}}$.

As per the released energy, we can use Equation S15 to rewrite the energy released from state (i) to (ii)

$$\begin{aligned}
\Delta U^*_{\mathrm{(ii)}} &= -\int_{\Delta V_{1\mathrm{(i)}}}^{\Delta V_{1\mathrm{(ii)}}} p_1 \mathrm{d}V - \int_{\Delta V_{2\mathrm{(i)}}}^{\Delta V_{2\mathrm{(ii)}}} p_2 \mathrm{d}V \\
&= -\int_{\Delta V_{1\mathrm{(i)}}}^{\Delta V_{1\mathrm{(iii)}}} p_1 \mathrm{d}V - \int_{\Delta V_{1\mathrm{(iii)}}}^{\Delta V_{1\mathrm{(ii)}}} p_1 \mathrm{d}V - \int_{\Delta V_{2\mathrm{(i)}}}^{\Delta V_{2\mathrm{(ii)}}} p_2 \mathrm{d}V - \int_{\Delta V_{2\mathrm{(i)}}}^{\Delta V_{2\mathrm{(iii)}}} p_{\mathrm{i}} \mathrm{d}V + \int_{\Delta V_{2\mathrm{(i)}}}^{\Delta V_{2\mathrm{(iii)}}} p_{\mathrm{i}} \mathrm{d}V \\
&= \left(-\int_{\Delta V_{1\mathrm{(i)}}}^{\Delta V_{1\mathrm{(iii)}}} p_1 \mathrm{d}V - \int_{\Delta V_{2\mathrm{(i)}}}^{\Delta V_{2\mathrm{(iii)}}} p_{\mathrm{i}} \mathrm{d}V \right) + \left(-\int_{\Delta V_{1\mathrm{(iii)}}}^{\Delta V_{1\mathrm{(ii)}}} p_1 \mathrm{d}V - \int_{\Delta V_{2\mathrm{(i)}}}^{\Delta V_{2\mathrm{(ii)}}} p_2 \mathrm{d}V + \int_{\Delta V_{2\mathrm{(i)}}}^{\Delta V_{2\mathrm{(iii)}}} p_{\mathrm{i}} \mathrm{d}V \right) \\
&= \Delta U^*_{\mathrm{(iii)}} + \left(\int_{\Delta V_{2\mathrm{(ii)}}}^{\Delta V_{2\mathrm{(iii)}}} p_{\mathrm{i}} \mathrm{d}V - \int_{\Delta V_{1\mathrm{(iii)}}}^{\Delta V_{1\mathrm{(ii)}}} p_1 \mathrm{d}V + \int_{\Delta V_{2\mathrm{(i)}}}^{\Delta V_{2\mathrm{(ii)}}} p_{\mathrm{i}} \mathrm{d}V - \int_{\Delta V_{2\mathrm{(i)}}}^{\Delta V_{2\mathrm{(ii)}}} p_2 \mathrm{d}V \right)
\end{aligned} \quad \text{(S16)}$$

where $\Delta U^*_{\text{(ii)}}$ is the released energy from state (i) to (ii), $\Delta U^*_{\text{(iii)}}$ is the released energy from state (i) to (iii). Here the snapping pressure of the spherical cap p_{i} is a constant, while p_1 and p_2 are function of the volume change of each shells (blue lines in Figure S15).

Since the total volume change is constant during snapping, according to Equation S10 we have

$$\Delta V_{1\text{(i)}} + \Delta V_{2\text{(i)}} = \Delta V_{1\text{(ii)}} + \Delta V_{2\text{(ii)}} = \Delta V_{1\text{(iii)}} + \Delta V_{2\text{(iii)}} \tag{S17}$$

The first two terms in the bracket of the last row of Equation S16 are

$$\begin{aligned} \int_{\Delta V_{2\text{(ii)}}}^{\Delta V_{2\text{(iii)}}} p_{\text{i}} \mathrm{d}V - \int_{\Delta V_{1\text{(iii)}}}^{\Delta V_{1\text{(ii)}}} p_1 \mathrm{d}V &= p_{\text{i}} \left(\Delta V_{2\text{(iii)}} - \Delta V_{2\text{(ii)}} \right) - \int_{\Delta V_{1\text{(iii)}}}^{\Delta V_{1\text{(ii)}}} p_1 \mathrm{d}V \\ &= p_{\text{i}} \left(\Delta V_{1\text{(ii)}} - \Delta V_{1\text{(iii)}} \right) - \int_{\Delta V_{1\text{(iii)}}}^{\Delta V_{1\text{(ii)}}} p_1 \mathrm{d}V \\ &= \int_{\Delta V_{1\text{(iii)}}}^{\Delta V_{1\text{(ii)}}} p_{\text{i}} \mathrm{d}V - \int_{\Delta V_{1\text{(iii)}}}^{\Delta V_{1\text{(ii)}}} p_1 \mathrm{d}V \end{aligned} \tag{S18}$$

Since $\Delta V_{1\text{(ii)}} < \Delta V_{1\text{(iii)}}$ and $p_{\text{i}} \geq p_1$, we have

$$\int_{\Delta V_{2\text{(ii)}}}^{\Delta V_{2\text{(iii)}}} p_{\text{i}} \mathrm{d}V - \int_{\Delta V_{1\text{(iii)}}}^{\Delta V_{1\text{(ii)}}} p_1 \mathrm{d}V = \int_{\Delta V_{1\text{(iii)}}}^{\Delta V_{1\text{(ii)}}} p_{\text{i}} \mathrm{d}V - \int_{\Delta V_{1\text{(iii)}}}^{\Delta V_{1\text{(ii)}}} p_1 \mathrm{d}V < 0 \tag{S19}$$

Similarly since $\Delta V_{2\text{(i)}} > \Delta V_{2\text{(ii)}}$ and $p_{\text{i}} \geq p_2$, the last two terms in the bracket of the last row of Equation S16 satisfies

$$\int_{\Delta V_{2\text{(i)}}}^{\Delta V_{2\text{(ii)}}} p_{\text{i}} \mathrm{d}V - \int_{\Delta V_{2\text{(i)}}}^{\Delta V_{2\text{(ii)}}} p_2 \mathrm{d}V < 0 \tag{S20}$$

Finally, Substituting Equation S19 and S20 into S16, we have

$$\Delta U^*_{\text{(ii)}} < \Delta U^*_{\text{(iii)}} \tag{S21}$$

The above demonstrate that $\Delta U^*_{\text{upper}} = \Delta U^*_{\text{(iii)}}$ is the upper bound of the released energy. State (iii) therefore describe the upper bounds of both volume change and released energy, which are theoretical limits fully determined by the spherical shell. The volume change and released

energy output of a given bi-shell valve can never surpass them, because the inflation of the imperfect shell during snapping can only cause a decrease in pressure.

S8. Flow chart of the design process

Figure S16 shows the two-steps approach here proposed for the design of our bi-shell valve. It involves the 4 metrics introduced in the main text of the paper: the upper bounds of volume and energy ($\Delta V^*_{\text{upper}}$ and $\Delta U^*_{\text{upper}}$) and the attainable ranges of output (ΔV^* and ΔU^*) within their respective bounds.

The focus of the first step is on the spherical cap only, and aims at identifying the upper bounds ($\Delta V^*_{\text{upper}}$ and $\Delta U^*_{\text{upper}}$) of the valve output. These quantities set the performance limits imposed by the spherical cap to a bi-shell system with any geometric parameters of the imperfect shell. With these upper bounds, we can select the geometry of a spherical cap (t_1 / R and h / R) that meet the requirements of volume and energy output prescribed by a given application.

In the second step, the emphasis shifts to the imperfect shell, and the goal is to obtain the attainable ranges of volume change ΔV^* and released energy ΔU^* for a bi-shell valve with the spherical cap selected in the first step. We do so by first exploring the design space of the imperfect shell. Then, we complete the valve design by selecting a set of the imperfect shell parameters, i.e. t_2 / R, θ_{U} and θ_{L}, that can meet the valve output requirements of the application (ΔV^* and ΔU^*). If there is no feasible design for the imperfect shell, we then return to the first step to revise the design of the spherical cap. With this approach, we can ensure to fully tap into the full potential of both shells and obtain a valve output that is close to the achievable maximum.

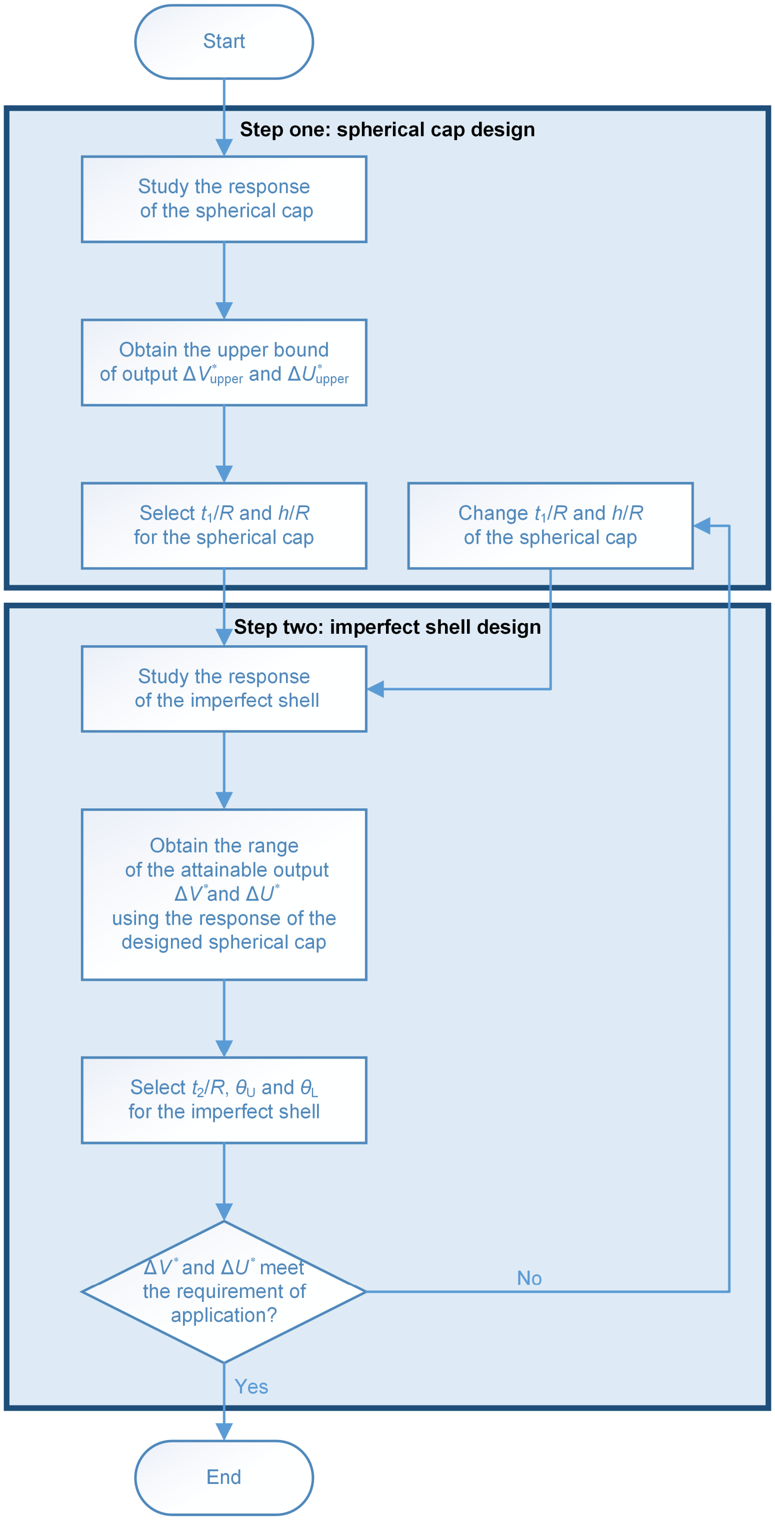

Figure S16. Design flowchart describing the steps to design the bi-shell valve.

S9. Alternative designs of the bi-shell valve

The bi-shell valve introduced in the main text operates through deflation. Here we introduce two design variations to achieve alternative functions: a *pneumatic volume fuse* and a *rapid inflation valve*.

- Figure S17A shows a *pneumatic volume fuse*. This concept modifies the original bi-shell valve operating in a deflation mode in the position of the output chamber, which is here moved on the top of the imperfect shell. In this configuration, before snapping, the imperfect shell can be deflated to generate a continuous volume output ΔV_2 (Figure S17B). When the imperfect shell is in the pre-snapping state (i), further deflation will trigger the snapping of the volume fuse, which reduces the volume change of the imperfect shell from state (i) to state (ii). The outcome is a pneumatic fuse: the volume change of the imperfect shell at state (i) sets the threshold of volume output that the fuse cannot exceed.
- Figure S17C shows a *rapid inflation valve*. The original bi-shell valve concept is here altered by flipping the two elastic shells upside down. This valve works in inflation mode in a way similar to that of the original valve that operation in a deflation mode. When slowly inflated at the inlet (Figure S17D), the imperfect shell first inflates to store energy and volume change. Upon snapping, the imperfect shell deflates from state (i) to state (ii) so as to release energy and volume change, while the spherical cap snaps upward. The advantage of this design is the provision of a fast volume output for the rapid inflation of any actuator that may be connected to the outlet.

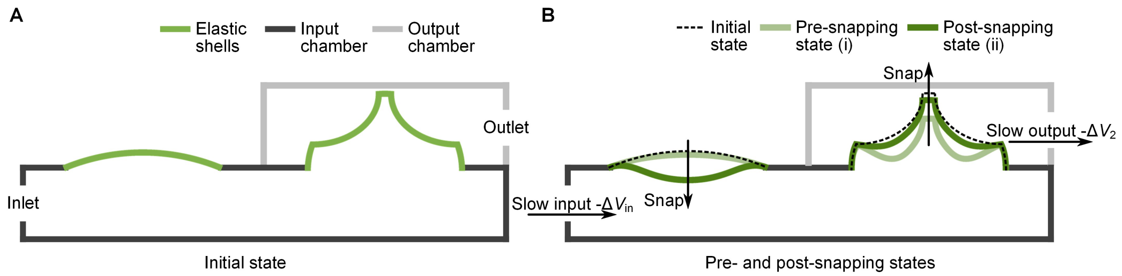

Figure S17. Alternative valve designs. (A) and (B) pneumatic volume fuse. (C) and (D) rapid inflation valve.

S10. Effect of elastic modulus on the performance of the bi-shell valve

To study the effect of material elasticity on the performance of the bi-shell valve, we perform a set of numerical simulations, where the shell material is assumed linear elastic with a Young's modulus ranging between 1 and 10 MPa. The geometry of the spherical cap and the imperfect shell are defined as: $R = 25$ mm, $t_1 / R = t_2 / R = 0.05$, $h / R = 0.2$, $\theta_L = 20°$, and $\theta_U = 85.9°$.

In Figure S18A, the pressure increases linearly with the Young's modulus, while the volume change is not affected by a change in the Young's modulus values. In Figure S18B, the released energy of the bi-shell valve increases linearly with the Young's modulus from 1 to 10 MPa with discrete step of 1 MPa. On the other hand, the volume output of the valve is not affected by a change in the Young's modulus values (Figure S18C).

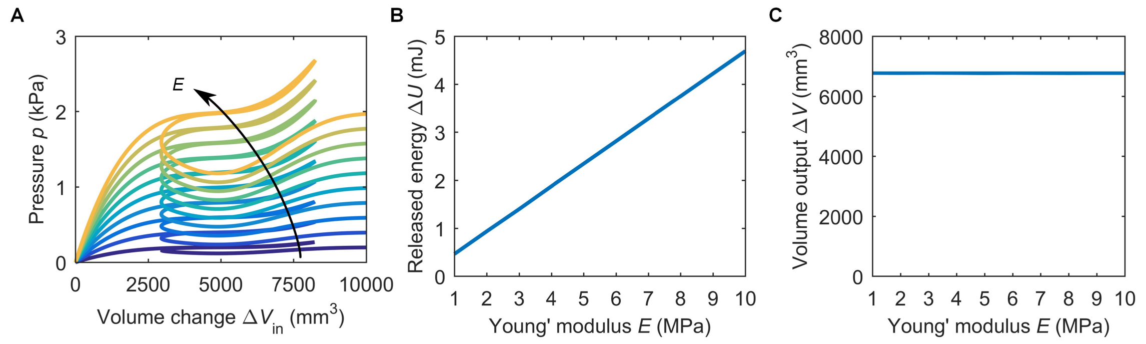

Figure S18. Influence of elastic modulus. (A) Pressure-volume change curves, (B) released energy ΔU , and (C) volume output ΔV of bi-shell valves with Young's modulus E increasing from 1 to 10 MPa with discrete step of 1 MPa.

S11. Integration of the bi-shell valve integrated into a soft robot or actuator.

To integrate our valve within a soft robot, the output chamber can be merged with the interior of the soft actuator, while the whole system of the bi-shell valve and the soft actuator can be controlled from the input chamber. A possible layout of the valve-actuator integration is given in Figure S19. Both the rigid input and output chambers can be replaced with thick soft walls by molding [6]. The integration would only require the merging of both molds, that of the bi-shell valve and that of the soft actuator. The exterior of the robot is purposely left as undefined because it can be shaped by design to deliver a certain function. For example to achieve motion, the exterior body can be designed as a partially corrugated cylinder with grips in contact with the ground.[6]

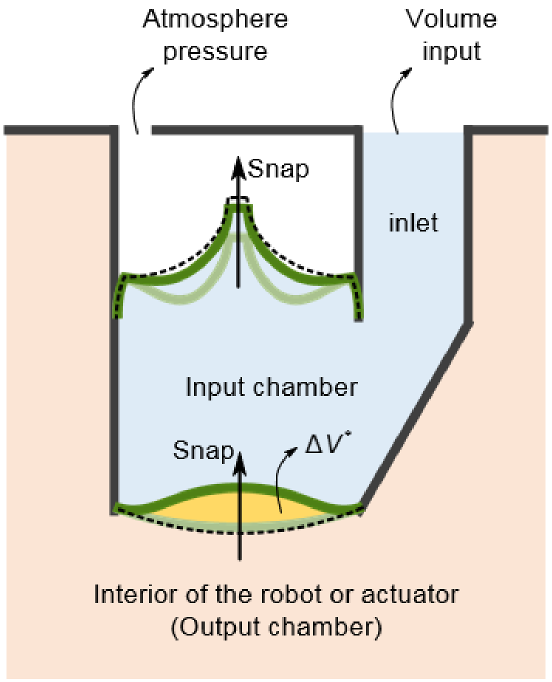

Figure S19. Schematic of the bi-shell valve integrated into a soft robot or actuator.

S12. Scenario of a similar valve with only one shell

If there is only the spherical cap (Figure S20), there can be no fast transfer of air volume between shells. This means that the total volume of the input and output chambers remains constant. As a result, the flow rate provided at the valve input equals that of the valve output. In our experiment, the flow rate at the valve inlet is 3 mL/min, a value that cannot provide fast actuation. On the other hand, if only the imperfect shell is present, fast actuation cannot yet be achieved. The reason is as for the above. No fast transfer of air volume between shells can occur, hence we cannot convert a low flow rate (input) to a fast flow rate (output).

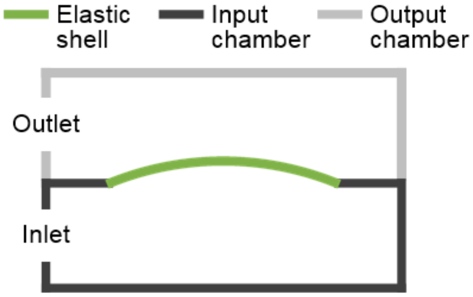

Figure S20. Scenario with only the spherical cap.

References

[1] A. Lee, F. López Jiménez, J. Marthelot, J. W. Hutchinson, P. M. Reis, *J. Appl. Mech.-Trans. ASME* **2016**, 83, 111005.
[2] C. Qiao, L. Liu, D. Pasini, *J. Mech. Phys. Solids* **2020**, 141, 103959.
[3] M. Pezzulla, S. A. Shillig, P. Nardinocchi, D. P. Holmes, *Soft Matter* **2015**, 11, 5812.
[4] J. W. Hutchinson, *Proc. R. Soc. A-Math. Phys. Eng. Sci.* **2016**, 472, 20160577.
[5] J. Paulose, D. R. Nelson, *Soft Matter* **2013**, 9, 8227.
[6] P. Rothemund, A. Ainla, L. Belding, D. J. Preston, S. Kurihara, Z. Suo, G. M. Whitesides, *Sci. Robot.* **2018**, 3, 7986.

Movie. S1. Snapping of the bi-shell valve.

Movie. S2. Fast pushing a table tennis ball with a striker actuated through the bi-shell valve.

Movie. S3. Actuation of the striker through the bi-shell valve.

Movie. S4. Actuation of the striker without the bi-shell valve.

Movie. S5. Reversible snapping of the bi-shell valve.
